# Time-resolved quantification of fine hand movements as a proxy for evaluating bradykinesia-induced motor dysfunction

**DOI:** 10.1038/s41598-024-55862-4

**Published:** 2024-03-04

**Authors:** Rachel K. Spooner, Bahne H. Bahners, Alfons Schnitzler, Esther Florin

**Affiliations:** 1 Institute of Clinical Neuroscience and Medical Psychology, Medical Faculty and University Hospital Düsseldorf, Heinrich Heine University, Düsseldorf, Germany; 2 Department of Neurology, Center for Movement Disorders and Neuromodulation, Medical Faculty and University Hospital Düsseldorf, Heinrich Heine University, Düsseldorf, Germany

**Keywords:** Accelerometer, Single-trial, Finger-tapping, Pronation-supination, Bradykinesia, Parkinson’s disease, Movement disorders, Motor control, Predictive markers

## Abstract

Bradykinesia is a behavioral manifestation that contributes to functional dependencies in later life. However, the current state of bradykinesia indexing primarily relies on subjective, time-averaged categorizations of motor deficits, which often yield poor reliability. Herein, we used time-resolved analyses of accelerometer recordings during standardized movements, data-driven factor analyses, and linear mixed effects models (LMEs) to quantitatively characterize general, task- and therapy-specific indices of motor impairment in people with Parkinson’s disease (PwP) currently undergoing treatment for bradykinesia. Our results demonstrate that single-trial, accelerometer-based features of finger-tapping and rotational hand movements were significantly modulated by divergent therapeutic regimens. Further, these features corresponded well to current gold standards for symptom monitoring, with more precise predictive capacities of bradykinesia-specific declines achieved when considering kinematic features from diverse movement types together, rather than in isolation. Herein, we report data-driven, sample-specific kinematic profiles of diverse movement types along a *continuous* spectrum of motor impairment, which importantly, preserves the temporal scale for which biomechanical fluctuations in motor deficits evolve in humans. Therefore, this approach may prove useful for tracking bradykinesia-induced motor decline in aging populations the future.

## Introduction

Bradykinesia or the general slowing of body movements, is a behavioral manifestation commonly observed in age-related pathologies (e.g., Parkinson’s disease (PD), HIV-infection, dystonia, progressive supranuclear palsy (PSP), multiple system atrophy (MSA), healthy aging^1–6^), and contributes to functional dependencies and poor quality of life in individuals. For example, bradykinesia-induced decrements in fine motor control of the body is the most pertinent symptom for probable PD diagnoses in aging cohorts, causing individuals to lose their ability to perform rudimentary activities of daily living (e.g., handwriting, brushing teeth, buttoning a shirt). Further, these deficits may contribute to the substantial individual, societal and economic burden of bradykinesia-induced motor deficits for people with PD (PwP) worldwide^1,2^. Currently, clinicians and investigators primarily use standard clinical assessments (e.g., Movement Disorder Society Unified Parkinson’s Disease Rating Scale (MDS-UPDRS) Part-III Motor Examination^7^) to *categorize* motor features of bradykinesia and their severity, although this approach has its limitations, as it largely relies on subjective rater designations, often leading to relatively poor reliability and reproducibility.^8–10^.

To date, there have been several methods proposed to *quantify* bradykinesia symptoms in healthy and clinical populations, some of which include the real-time monitoring of movement kinematics during standardized movements using low-cost wearable sensor devices (e.g., accelerometers, gyroscopes)^10–14^. While these studies have provided invaluable insight regarding the general progress of motor decline observed in individuals, the majority of these studies report time-averaged (e.g., averaged over trials, task blocks, or time over minutes/hours) metrics of relevant behavioral or clinical features (e.g., movement amplitude, movement speed, movement pacing). However, this approach may obscure important time-dependent variations in symptom manifestations that are important clinical indicators of motor dysfunction (e.g., MDS-rated time-dependent decrement in movement amplitude and/or frequency, time-dependent presence of hesitations, etc^7^). Moreover, the movement paradigms used to quantify bradykinesia symptoms varied widely across the aforementioned studies (e.g., MDS-rated movement protocols and laboratory tasks^14–21^, self-paced or naturalistic movements^22–24^), making comparison and interpretation of quantitative, albeit time-averaged movement features, especially difficult across studies and clinical cohorts. Interestingly, Stamatakis et al. (2013) proposed a method for quantifying single-trial event dynamics from accelerometer traces during finger tapping protocols and could demonstrate relatively good predictive performance of expert-rated UPDRS sub-scores from accelerometer-based features of tapping (model accuracy > 0.95)^11^. However, it is currently unclear how time-resolved features of movement relate to relevant clinical statuses of the patient (e.g., therapeutic regimen, disease progression, age of onset, etc.)—which will be of utmost importance when considering the translational efficacy of objective, sensor-based symptom quantification methods for individuals exhibiting motor dysfunction.

**Figure 1:**
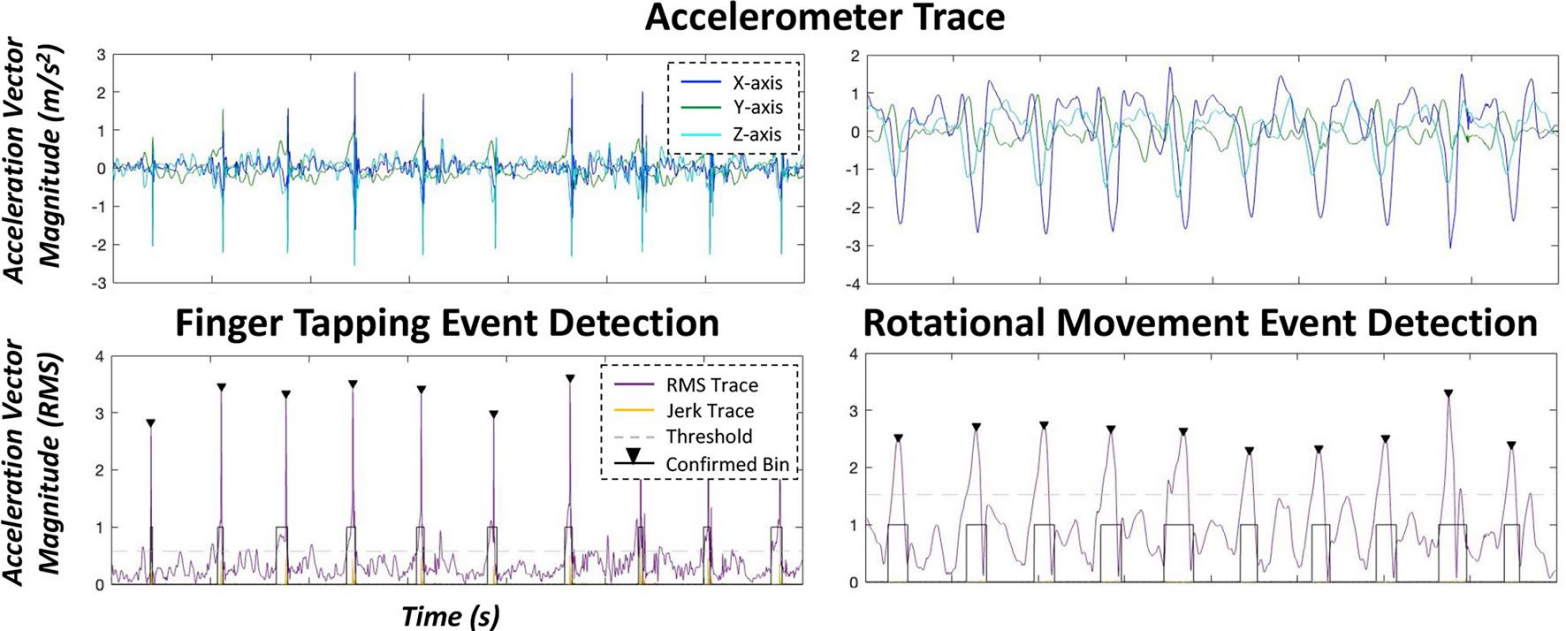
**Systematic Detection of Finger-Tapping and Rotational Hand Movement Metrics.** Exemplary definition of a patient’s single-trial finger-tapping (left panel) and rotational hand movement (right panel) windows using a fixed threshold algorithm based on root mean square (i.e., general acceleration magnitude (RMS)) and jerk (i.e., rate of change of acceleration) percentile thresholds (bottom panel) derived from preprocessed accelerometer data (top panel). Axis labels are fixed for all graphs.

Thus, the goal of the current proof-of-concept study was to develop a data-driven, time-resolved quantification of fine motor hand function reflective of bradykinesia-induced impairment using standardized movement protocols (i.e., UPDRS Item 3.4 and Item 3.6) and sensor-based monitoring of movement kinematics (i.e., triaxial accelerometers, Fig. 1). As bradykinesia is a cardinal hallmark of PD and pertinent for probable PD diagnoses, we chose to enroll a cohort of PwP undergoing current treatment (i.e., pharmacological intervention and deep brain stimulation (DBS)) for bradykinesia. Furthermore, as we aimed to characterize precise symptom (i.e., movement type) *and* disease-related (i.e., medication and stimulation status) alterations in quantitative motor outcomes, we enrolled PwP implanted with subthalamic deep brain stimulation (STN-DBS) in addition to their levodopa treatment, as STN-DBS is a highly effective treatment for alleviating bradykinesia symptoms in PwP. Moreover, more substantial mechanistic and clinical insights are guaranteed from this cohort, as the direct modulation of neural networks (and subsequent behavioral outcomes) is known given the chosen stimulation site. Using factor analyses of multidimensional movement features and linear mixed effects models (LME), we aimed to evaluate the predictive capacity of quantitative kinematic profiles of hand movements for characterizing divergent clinical statuses and bradykinesia-specific symptoms in PwP.

## Results

### Single-trial movement dynamics index relevant clinical statuses in PwP

In order to interrogate the behavioral impact of diverging clinical statuses in PwP (i.e., presence or absence of medication and stimulation), we examined single-trial accelerometer metrics of finger-tapping and rotational hand movements separately using LMEs with clinical status as a fixed effect and subject and trial number as a nested random effect (Figs. S1, S2).

In regard to finger-tapping event metrics, we observed significant main effects of clinical status on tap acceleration (F(1207.9) = 31.37, *p* < 0.001), acceleration variability (F(1266.1) = 65.74, *p* < 0.001), inter-tap interval (ITI i.e., tapping pace: F(1226.1) = 25.96, *p* < 0.001), ITI variability (F(1227) = 5.78, *p* = 0.001), movement execution smoothness (i.e., acceleration jerk: F(1230.8) = 6.59, *p* < 0.001), and acceleration jerk variability (F(1279.7) = 17.48, *p* < 0.001). In regard to tap acceleration, post-hoc analyses revealed that single-trial tap acceleration was faster when participants were tested during clinically effective therapeutic regimens (i.e., stim ON/med ON) compared to stim OFF/med OFF (t(950) = -8.98, *p* < 0.001), stim OFF/med ON (t(934) = − 6.32, *p* < 0.001), and stim ON/med OFF configurations (t(928) = − 7.40, p < 0.001; Table S2 and Fig. S1). Similarly, we observed a step-wise reduction in intra-tap acceleration variability (reflective of more consistent intra-tap acceleration magnitudes) with increased efficacy in clinical regimens (i.e., from stim OFF/stim OFF, stim OFF/med ON, stim ON/med OFF, to stim ON/med ON; *ps* < 0.004, see Fig. S1 and Table S2 for full model results). In regard to pacing-related metrics (i.e., ITI, ITI variability), there was a significant main effect of clinical status, such that ITIs were shorter (i.e., faster tapping pace) and less variable (i.e., more consistent tapping pace) during effective therapies (i.e., stim ON/med ON) compared to less-effective therapies (*ps* < 0.032; see Fig. S1 and Table S3 for full model results). Post-hoc testing revealed that movement execution smoothness (i.e., rate of change in acceleration: jerk in m/s^3^) was higher, and jerk variability (in %) was lower, reflective of smoother index-to-thumb execution-related tapping dynamics during more clinically effective therapies (stim ON/med ON) vs. less-effective ones (stim ON/med OFF, stim OFF/med ON and stim OFF/med OFF; *ps* < 0.041, see Fig. S1 and Table S4 for full model results). Finally, we observed no significant effect of therapeutic status on changes in finger tapping acceleration magnitudes over time (i.e., improvements or decrements in acceleration; F(51.5) = 2.68, *p* = 0.057; Table S5), nor on changes in movement pacing over time (i.e., improvements or decrements in movement pacing; F(50.5) = 1.28, *p* = 0.292; Table S6).

Concerning rotational movement kinematics, we observed significant main effects of clinical status on rotation acceleration (F(953.7) = 94.77, *p* < 0.001), acceleration variability (F(1005.9) = 14.13, p < 0.001), movement execution smoothness (i.e., acceleration jerk: F(946.0) = 2.72, *p* = 0.043) and acceleration jerk variability (F(989.4) = 18.08, *p* < 0.001), albeit trending effects were observed for pacing-related kinematic features of rotational hand movements (inter-rotation interval: IRI: F(955.9) = 2.54, *p* = 0.056; IRI variability: F(955.6) = 2.23, *p* = 0.084). Post-hoc analyses of acceleration-based features of rotational hand movements were readily distinguishable across divergent clinical states (*ps* < 0.005, for full model results see Table S7 and Fig. S2), with more clinically effective therapies generally yielding faster intra-rotation accelerations, which were also more consistent (i.e., lower CV) with stimulation and medication. Similar to finger-tapping dynamics, post-hoc testing revealed that movement execution smoothness was higher and less variable during more clinically effective therapies (stim ON/med ON) vs. less-effective ones (stim OFF/med OFF for jerk: t(732) = − 2.67, *p* = 0.039; and jerk variability: t(770) = − 4.24, *p* < 0.001; for full model results, see Table S9 and Fig. S2). Finally, we observed no significant effect of therapeutic status on changes in rotational movement acceleration magnitudes over time (i.e., improvements or decrements in acceleration; F(48.7) = 0.25, *p* = 0.863; Table S10), nor on changes in movement pacing over time (i.e., improvements or decrements in movement pacing; F(64) = 0.24, *p* = 0.867; Table S11) (Fig. 1).

### Empirically-derived movement profiles predict clinical evaluations of motor impairment

**Figure 2:**
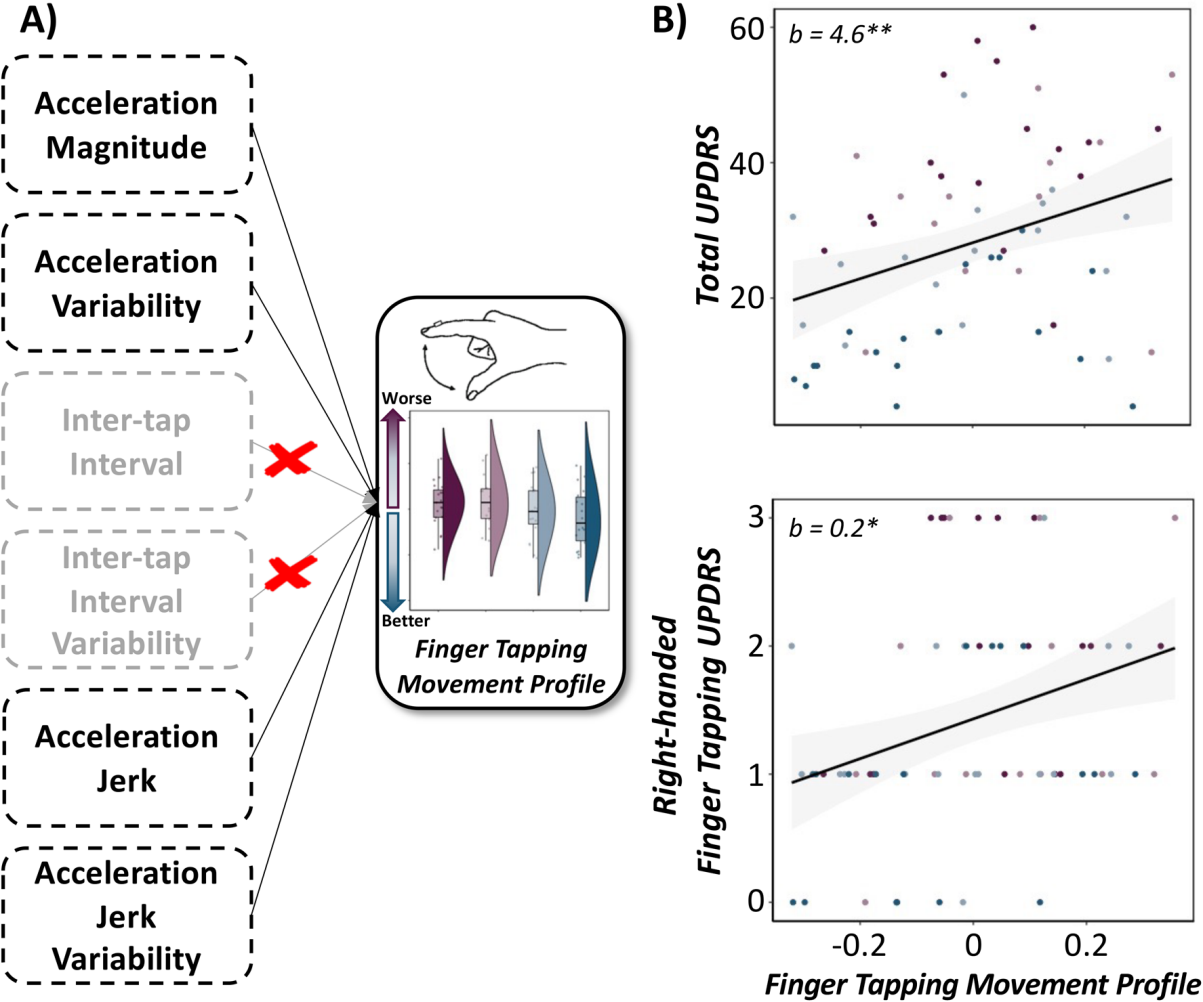
**Quantitative Finger-Tapping Movement Profiles Predict Clinical Outcomes in PD.** (Left) Conceptual model of the principal components analysis used to derive a single finger-tapping movement profile per patient based on metrics exhibiting significant alterations as a function of medication and DBS stimulation status (i.e., acceleration magnitude, acceleration variability, acceleration jerk, and jerk variability), with lower values indicative of better movement profiles. (Right): Linear mixed effects models of finger-tapping movement profiles on total (top) and right-handed finger-tapping (bottom) UPDRS scores, separately. Lower movement profile scores (i.e., better behavioral performance) were predictive of lower UPDRS total and sub-scores (i.e., less severe motor deficits) regardless of medication and stimulation status and controlling for symptom laterality. X-axes are fixed for each scatterplot. 95% confidence intervals are displayed in gray for each regression line. *p_*corr*_ < .05, **p_*corr*_ < .01. b = unstandardized beta coefficient.

Next, we aimed to evaluate the predictive capacity of empirically-derived, sample-specific movement profiles on traditional clinical evaluations of motor symptom severity (i.e., UPDRS Part III total and Item 3.4/3.6 sub-scores) in our participants. As described in the methods and in our previous work^25^, we constructed a movement profile score pertinent to finger-tapping and rotational hand movements, separately, using an exploratory factor analysis (EFA) of a compilation of accelerometer metrics exhibiting significant alterations as a function of clinical status. These included acceleration magnitude, acceleration variability, inter-movement interval, inter-movement interval variability, acceleration jerk, jerk variability, slope of acceleration magnitude (i.e., acceleration improvement/decrement over time) and slope of movement frequency (i.e., inter-movement interval improvement/decrement over time). The initial EFA based on all eight features during finger-tapping indicated a single-factor solution with poor fit (χ^2^ = 60.19; RMSEA = 0.18; 90% CI [0.13, 0.23]; CFI = 0.81; SRMR = 0.09). Since pacing-related kinematic features and kinematic decrement features loaded less strongly onto the factor (λs = 0.66 for ITI, 0.67 for ITI variability, 0.06 for acceleration magnitude decrement, 0.01 for inter-movement interval decrement, respectively), sequentially excluding these variables yielded a single-factor solution with excellent model fit (χ^2^ = 7.81; RMSEA = 0.20; 90% CI [0.07, 0.36]; CFI = 0.96; SRMR = 0.05). Thus, our empirically-defined quantification of finger-tapping movement profiles was comprised of reverse coded acceleration magnitude, acceleration variability, reverse coded acceleration jerk, and jerk variability, which accounted for 68.8% of the variance in finger-tapping movement profiles in our sample. Of note, lower finger-tapping movement profiles are reflective of better behavioral performance, and these scores were used as a fixed effect (continuous variable) in subsequent LMEs. For supplementary analyses of finger tapping movement profiles as a function of therapeutic status, see Table S14. Specifically, LMEs of finger-tapping movement profiles on total UPDRS Part-III and right-handed finger-tapping UPDRS Item 3.4 sub-scores suggested that lower movement profile scores (i.e., reflective of better behavioral performance) were significantly predictive of lower UPDRS Part-III total (*F*(42.2) = 6.06, *p* = 0.018) and Item 3.4 sub-scores (*F*(56.9) = 4.72, *p* = 0.034; i.e., less severe general and item-specific motor deficits) regardless of medication and stimulation status and above and beyond symptom laterality indices (Fig. 2). Moreover, we observed a significant main effect of symptom laterality on UPDRS Part-III total (F(60.4) = 15.88, p < 0.001) and item-specific scores (F(64.7) = 43.06, *p* < 0.001), such that individuals exhibiting more right-lateralized symptoms (i.e., negative symptom laterality index) had more severe total and item-specific motor deficits. Finally, we observed no significant finger tapping movement profile x symptom laterality interaction on UPDRS total or item-specific sub-scores (*ps* > 0.417).

**Figure 3:**
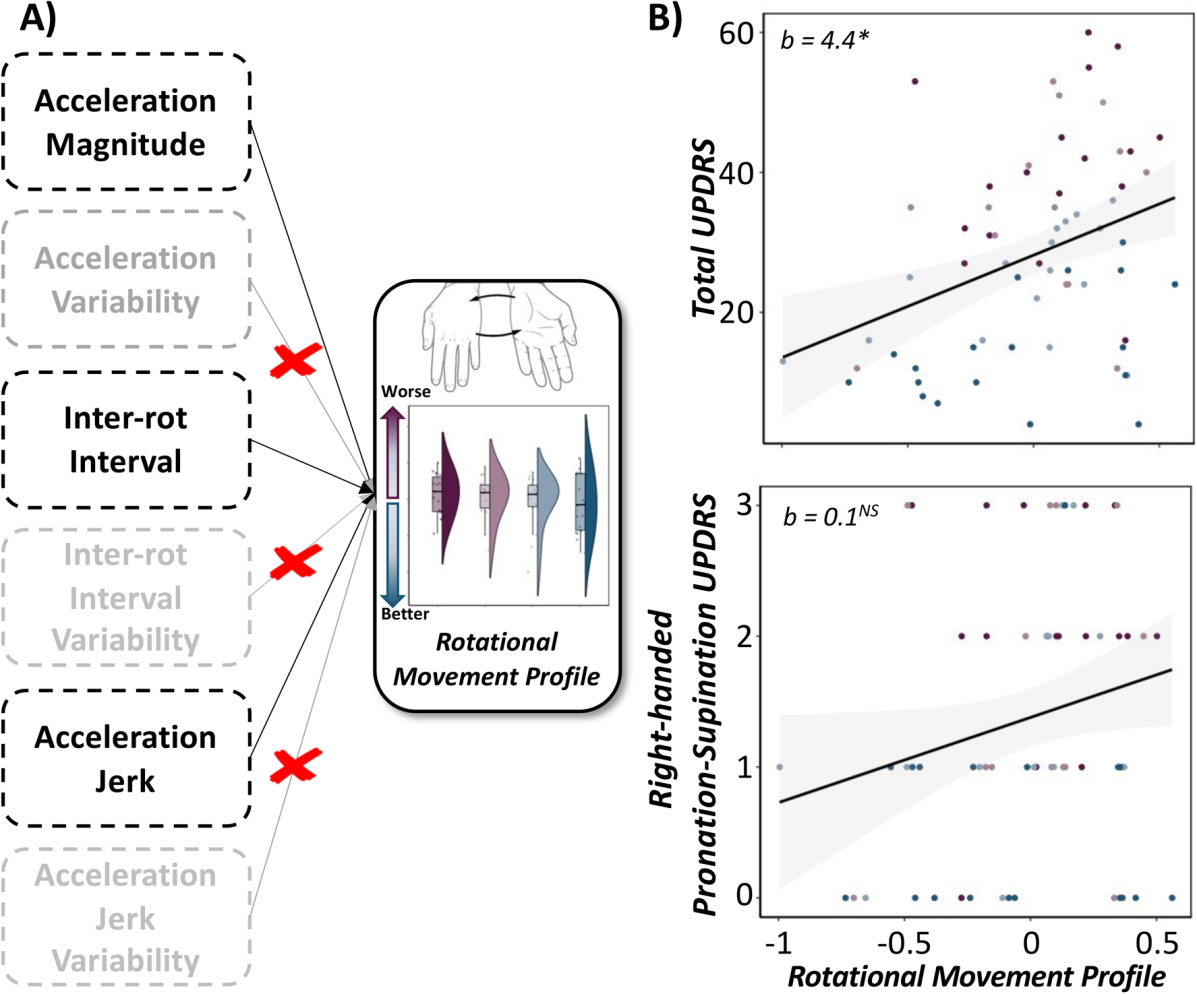
**Quantitative Rotational Movement Profiles Predict Clinical Outcomes in PD.** (Left) Conceptual model of the principal components analysis used to derive a single rotational movement profile per patient based on metrics exhibiting significant alterations as a function of medication and DBS stimulation status (i.e., acceleration magnitude, acceleration jerk, inter-rotation interval), with lower values indicative of better movement profiles. (Right): Linear mixed effects models of rotational movement profiles on total (top) and right-handed pronation-supination (bottom) UPDRS scores, separately. Lower movement profile scores (i.e., better behavioral performance) were predictive of lower UPDRS total and sub-scores (i.e., less severe motor deficits) regardless of medication and stimulation status and controlling for symptom laterality. X-axes are fixed for each scatterplot. 95% confidence intervals are displayed in gray for each regression line. *p_*corr*_ < .05. NS = not significant. b = unstandardized beta coefficient.

The initial EFA based on all eight features of rotational hand movements indicated a single-factor solution with poor fit (χ^2^ = 22.32; RMSEA = 0.04; 90% CI [0.00, 0.20]; CFI = 0.97; SRMR = 0.12). Since acceleration variability, IRI variability, jerk variability, slope of acceleration magnitude over time and slope of movement pacing over time loaded poorly onto the factor (λs = 0.52, 0.07, − 0.28, − 0.11, − 0.12, respectively), sequentially excluding these variables yielded a single-factor solution with excellent model fit (χ^2^ = 0.00; RMSEA = 0.00; 90% CI [0.00, 0.00]; CFI = 1.00; SRMR = 0.00). Our empirically-defined, sample-specific quantification of rotational movement profiles was comprised of reverse coded acceleration magnitude, reverse coded acceleration jerk, and IRI, which accounted for 63.3% of the variance in rotational movement profiles (Fig. 3). Of note, lower rotational movement profile scores are reflective of better behavioral performance and these scores were used as a fixed effect (continuous variable) in our LMEs. For supplementary analyses of rotational movement profiles as a function of therapeutic status, see Table S15.

Specifically, LMEs of rotational movement profiles on total UPDRS-III and right-handed, pronation-supination UPDRS Item 3.6 sub-scores suggested that lower movement profile scores (i.e., better behavioral performance) were significantly predictive of lower UPDRS Part-III total (F(60.1) = 7.04, *p* = 0.010), but not item-specific sub-scores (F(59.8) = 1.54, *p* = 0.219) regardless of medication and stimulation status and symptom laterality (Fig. 3). Moreover, we observed a significant main effect of symptom laterality on UPDRS total (F(60.1) = 14.97, *p* < 0.001) and rotational movement sub-scores (F(59.1) = 18.80, *p* < 0.001), such that individuals exhibiting more right-dominant motor symptoms (i.e., negative symptom laterality index) had more severe total and item-specific UPDRS scores. Finally, we observed no significant rotational movement profile x symptom laterality interactions on clinical evaluations of motor impairment (*ps* > 0.765). Taken together, these data suggest that quantitatively-derived finger-tapping and rotational movement profiles, as measured in the current study correspond well to traditional clinical assessments of overall and task-specific symptom heterogeneity regardless of the therapeutic state of the participant, as well as the more affected side of motor impairment.

### Diverse hand movement kinematics differentially predict bradykinesia symptoms

**Figure 4:**
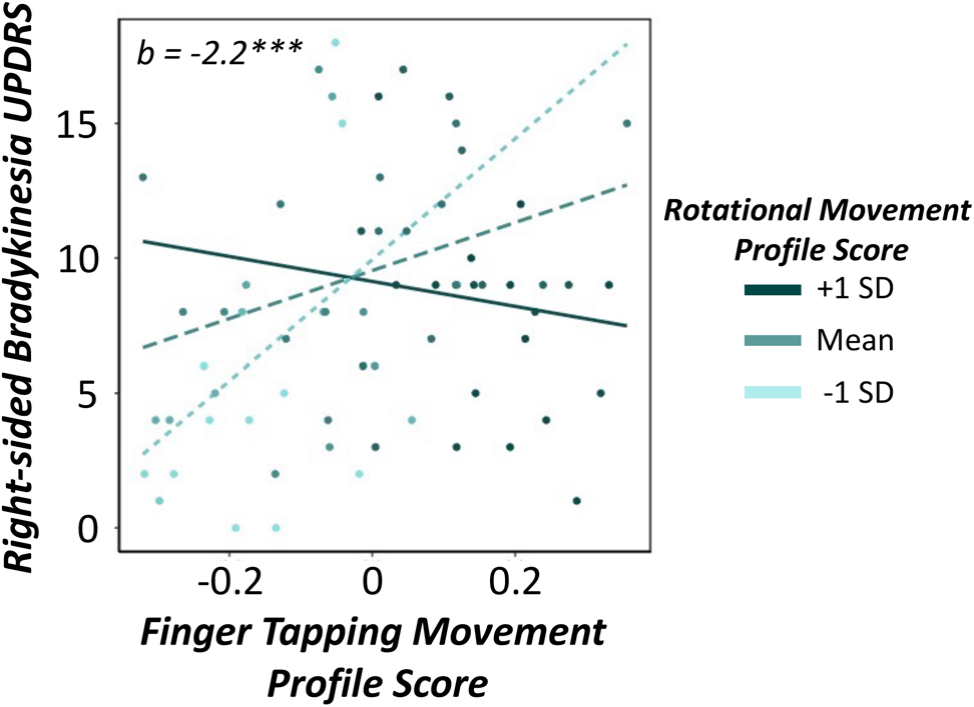
**Diverse Hand Movement Kinematics Differentially Predict Bradykinetic Symptoms in PD.** Linear mixed effects model of empirically-derived finger-tapping movement profiles (continuous), rotational movement profiles (continuous) and their interaction on right-sided bradykinesia UPDRS score (i.e., comprising the sum of Item 3.4, 3.5, 3.6, 3.7, 3.8 sub-scores, with lower values reflective of less severe bradykinesia symptoms) controlling for symptom laterality indices. Graphic denotes the relationship between finger-tapping movement profile scores on the x-axis and bradykinesia severity scores on the y-axis. Regression lines depicting the aforementioned relationships for lower and higher rotational movement profiles are based on ± 1 SD cutoffs of the mean rotational movement profile score (also displayed). These cutoffs were used for visualization purposes of the movement-type interaction effect only, as quantitative movement profile scores were treated as continuous variables for each movement type in the model. There was a significant interaction of movement type, such that during instances of better rotational movement performance (i.e., lower movement profile scores: − 1 SD), better finger-tapping performance was predictive of less severe bradykinetic symptoms in our cohort. SD: standard deviation. ***p_*corr*_ < .005. b = unstandardized regression coefficient.

Finally, we examined the differential predictive capacity of diverse movement types (i.e., finger-tapping movement profiles, rotational movement profiles, and their interaction) on bradykinesia-specific symptoms controlling for symptom laterality using LMEs in R. Of note, a right-sided bradykinesia UPDRS score (comprising the sum of Item 3.4, 3.5, 3.6, 3.7, 3.8 sub-scores) was used as a dependent variable in the subsequent analysis. Our results indicated a significant main effect of finger-tapping movement profiles on bradykinesia-specific symptoms (F(48.6) = 2.25, *p* = 0.044), such that lower finger-tapping movement profiles (i.e., better finger tapping performance) were predictive of lower bradykinesia scores, reflective of less severe bradykinesia-related symptoms in our cohort. In contrast, we observed no significant main effect of rotational movement profiles on bradykinesia-specific symptoms (F(54.0) = 0.25, *p* = 0.617), albeit we did observe a significant movement type interaction (F(65.8) = 13.35, *p* < 0.001), such that together, lower finger-tapping *and* rotational movement profile scores (i.e., quantifiable improvements in tapping and rotational movement kinematics) were stronger predictors of lower bradykinesia symptom severity scores than either movement type in isolation and above and beyond symptom laterality. Next, we observed a significant main effect of symptom laterality (F(65.2) = 44.82, *p* < 0.001), such that individuals with right-lateralized symptoms (i.e., negative symptom laterality indices) had more severe right-sided bradykinesia deficits. In addition, we observed significant movement profile x symptom laterality interactions, such that individuals with right-lateralized motor symptoms and higher finger tapping and rotational movement profiles (i.e., worse quantitative measures of behavioral performance), had more severe overall bradykinesia scores (F(59.1) = 5.93, b = − 1.62, *p* = 0.018 for finger tapping), while the opposite trajectory was observed for right-lateralized patients exhibiting worse behavior on rotational movement tasks (i.e., higher rotational movement profiles; F(66.0) = 6.99, b = 1.49, *p* = 0.010). Finally, finger-tapping and rotational movement dynamics, as well as symptom asymmetry indices together accounted for 70.2% of the variance in clinically-defined bradykinesia severity scores (Fig. 4).

## Discussion

In the current study, we applied advanced, time-resolved analyses to wearable sensor-based recordings of fine hand movements to determine the predictive capacity of quantitative kinematic features for characterizing clinically-relevant fluctuations in motor symptom severity (e.g., medication and stimulation regimens) in a cohort of PwP. Specifically, we observed that quantitative movement features observed at the single-trial level (e.g., execution-related movement acceleration, movement pacing, movement execution smoothness) were differentially modulated by diverging medication and stimulation states of the individual. Moreover, using empirically-derived definitions of task-specific motor impairment (i.e., finger-tapping and rotational movement profile scores) exhibiting significant therapeutic fluctuations and LMEs, our quantitative marker of motor symptom severity demonstrated good correspondence to traditional clinical evaluations of motor impairment, as evidenced by significantly predicting general, task-specific and bradykinesia-specific MDS-UPDRS outcomes, which importantly held above and beyond indices of symptom laterality (i.e., most affected side of motor impairment) in the current cohort. Below, we discuss the implications of these findings and the time-resolved methods used herein for indexing clinically-relevant fluctuations in motor performance in humans.

Our findings suggesting that time-resolved, quantitative features of fine motor hand function were differentially sensitive to changes in medication and stimulation states were not surprising when considering the immense heterogeneity of symptom presentations in PD. As no single individual presents with the same combination of motor impairment, this individual-level heterogeneity in motor outcomes is important to consider. Essentially, predominant symptom profiles of PwP (e.g., tremor-dominant, akinetic-rigid dominant) have been traced to at least partially dissociable neurophysiological substrates^26–28^, are differentially impacted by important clinical features (e.g., age of onset)^2,9,29–31^, and ultimately, are related to disparate disease prognoses and mortality rates^2,29,31^. For example, individuals with predominant akinetic-rigid symptom presentations (i.e., bradykinesia) may experience a faster or more severe progression of PD, leading to poorer long-term clinical outcomes such as severe declines in mobility, increased risk of falls due to increased postural instability, and even the development of cognitive impairment or dementia^2,29,31^. Moreover, individuals presenting with predominant right-lateralized motor symptoms may experience more severe and progressed disease states than those presenting with predominantly left-lateralized symptoms^32–34^. In the current study, finger-tapping and rotational hand movement dynamics related to general execution-related movement acceleration, movement pacing (i.e., inter-movement interval), movement execution smoothness (i.e., acceleration jerk) and the coefficient of variation in these metrics (reflecting the variability in movement features) often showed stepwise improvements in motor outcomes with increased clinical efficacy of therapeutic regimens administered (i.e., from stim OFF/med OFF to stim ON/med ON configurations). In contrast, outcomes related to changes in movement acceleration or pacing (i.e., acceleration/pacing improvements/decrements over time elapsed on the task) were unaffected by therapeutic regimens regardless of the movement performed. Interestingly, inter-trial variation of task-specific movement acceleration and movement smoothness (i.e., acceleration jerk variability), as opposed to pacing-related features (e.g., inter-movement interval and variability) of the movement block, could more effectively distinguish between each clinically-relevant medication and stimulation state for finger-tapping and rotational hand movement paradigms, respectively. Taken together, these data suggest that multidimensional, time-resolved features of movement kinematics during standardized clinical protocols may allow investigators to more precisely capture biomechanical *and* clinically-relevant nuances in motor function that may otherwise be difficult to rate using subjective, time-averaged ratings of motor deficits.

Our most important finding established a link between empirically-derived, task-specific movement profiles and traditional evaluations of motor symptom severity in PwP. Using factor analyses to construct a latent variable reflecting task-specific movement profile scores which were comprised of a compilation of accelerometer metrics reflecting relevant therapeutic effects (i.e., significant deviations as a function of medication/stimulation state), we observed that smoother, more controlled finger-tapping and rotational hand movements (i.e., greater acceleration magnitude and movement smoothness, lower variability in acceleration-related metrics for finger-tapping profiles; greater acceleration magnitude and movement smoothness, faster movement pace for rotational movement profiles) were predictive of less severe general and/or task-specific UPDRS outcomes of motor symptom severity (i.e., total and Item 3.4 UPDRS scores, respectively). Moreover, sample-specific definitions of finger-tapping performance were robust predictors of bradykinesia-specific UPDRS outcomes (i.e., right-sided bradykinesia sum score), while rotational hand movement profiles did not significantly relate to bradykinesia symptoms above and beyond the effects of finger-tapping performance alone. While it is interesting that rotational movements did not significantly relate to item- or bradykinesia-specific motor impairments in the current sample, this suggests that rotational movement kinematics are not robust predictors of PD-related motor impairment, as reported for finger tapping kinematics. This may be attributable, at least in part to the increased difficulty of completing whole hand pronation-supination movements compared to more simple, index-to-thumb finger taps, making subjective ratings of clinical impairment more cumbersome for patients and raters alike. Nevertheless, our results revealed a significant movement type interaction on bradykinesia symptom severity, such that better behavioral profiles during finger-tapping *and* rotational movement paradigms (i.e., lower movement profile scores) were predictive of less severe right-sided bradykinesia symptoms in our cohort of PwP and furthermore, accounted for over 70% of the variance in clinically-rated bradykinesia symptoms when controlling for symptom laterality (i.e., left-lateralized bradykinesia sum score—right lateralized bradykinesia sum score). Importantly, these findings suggest that quantitative movement profiles derived from accelerometer recordings of finger tapping and rotational hand movements correspond well to traditional clinical evaluations of motor impairment (i.e., relate to well-established, categorical definitions of motor symptoms and their severity) and furthermore, may be used *in concert with* clinical ratings to provide a more comprehensive understanding of the precise biomechanical aberrancies contributing to motor symptoms in PwP.

The aforementioned findings of quantitative and clinical outcomes are not surprising, as sensor-based analyses of finger-tapping performance as rated using Item 3.4 of the MDS-UPDRS have shown promise for characterizing bradykinesia-related impairments in PwP using regression- or machine learning-based predictions of UPDRS outcomes^11,12,35–37^. However, despite these methodological advancements and focus on extracting quantitative behavioral features from sensor-based recordings, these approaches are still limited to yielding a categorical, time-averaged definition of motor symptoms and their severity (i.e., predicted UPDRS sub-score of task block). As a result, herein, we report a data-driven, sample-specific kinematic profile of movement using a *continuous* spectrum of motor impairment, which importantly, can preserve the temporal scale (i.e., inter-movement variation in kinematic features per individual movement) for which biomechanical fluctuations in motor deficits may evolve. Importantly, this approach has several advantages, as it may allow for future decoding studies of behavioral deficits that track alongside other physiological aberrancies (e.g., weakened peri-movement beta and gamma oscillations in the basal ganglia-cortical loop^26,38–42^) in humans. Moreover, the interpretation of sample-specific, quantitative definitions of movement as described herein (i.e., latent variables from EFAs) may provide unique mechanistic insight regarding the precise kinematic features pertinent to bradykinesia symptom manifestation in the studied cohort (i.e., based on the magnitude and directionality of achieved loadings). For example, based on our analyzed cohort, we may presume that behavioral deficits in movement execution-related acceleration or smoothness, but not pacing, are greater indicators of general and task-specific motor dysfunction (i.e., total and Item 3.4 UPDRS scores, respectively) and bradykinesia-specific impairments in the current cohort, as evidenced by deviations in achieved loadings and superior model fit indices when including these kinematic features specifically. Thus, these features may be clinically-relevant for indexing PD-specific manifestations of bradykinesia and importantly, may prove to be effective targets for therapeutic interventions aiming to ameliorate specific functional declines (e.g., execution-related movement kinematics rather than pacing-related dynamics) in clinical populations in the future.

To conclude, this study developed a data-driven, quantitative characterization of fine motor hand function from triaxial accelerometer recordings of standardized movement protocols which corresponded well to traditional clinical evaluations of general, task-specific and bradykinesia-specific symptoms in a cohort of PwP. Specifically, we observed that inter-trial variation in execution-related movement acceleration and movement smoothness in particular (i.e., acceleration and jerk variability, respectively), were kinematic features most sensitive to distinguishing divergent therapeutic regimens in PwP (i.e., administration of dopaminergic medication and therapeutically-effective STN-DBS), which aligns well to prior work from our laboratory describing alterations in movement kinematics as a function of various parameter settings pertinent to STN-DBS clinical efficacy (e.g., DBS contact orientation or stimulation amplitude^25,37,43^). Furthermore, better finger-tapping and rotational hand movement profiles derived from accelerometer-based kinematics were differentially predictive of less severe bradykinesia-specific motor outcomes, suggesting that the quantitative evaluation of diverse movement types in concert, rather than in isolation, could provide more precise, comprehensive estimations of bradykinesia symptoms. Importantly, while our study benefited from adhering our movement paradigms to current clinical standards for finger-tapping and pronation-supination assessments in PwP to facilitate comparison with standardized movement protocols, future work relating task-specific fluctuations in motor deficits with at-home assessments of movement variability (e.g., task-irrelevant movement velocity over minutes or hours) will provide valuable insight regarding the generalizability of the aforementioned task-dependent kinematic features of motor impairment. Moreover, although the current study focused its investigation on PwP as a first proof-of-concept for indexing bradykinesia-related impairments, future work will undoubtably benefit from examining such quantitative movement profiles using similar standardized movements in atypical forms of parkinsonism and other neurological disorders exhibiting age-, disease- or therapy-related manifestations of bradykinesia (e.g., dystonia, PSP, MSA, Wilson’s disease, chorea, ataxia, HIV-infection^4,44–50^), in order to confirm its specificity for characterizing PD-related bradykinesia in particular.

While our results are promising, the study is not without its limitations. Of note, the data presented in the current study was collected from right hand movements of finger tapping and whole hand pronation-supination, as this data was collected as part of a larger DFG-funded study (TRR295—424778381) which utilized left-lateralized STN-DBS protocols during behavioral and neurophysiological recordings to reduce contamination of neurophysiological datasets by large ferromagnetic artifacts induced by the DBS cables and generator implanted on the right body half in our participants. Thus, it is possible that the kinematic features presented in the current study are not reflective of the most affected side of motor impairment. This is an important consideration, as prior work suggests that PwP exhibiting right-lateralized motor symptoms may experience more severe and progressed disease states than those presenting with motor symptoms on their left side^32–34^. Importantly, we controlled for symptom laterality in the current analyses of our quantitative movement profiles and total, item-specific, as well as bradykinesia-related impairments on the MDS-UPDRS by calculating a symptom laterality index subtracting right-sided bradykinesia scores from their left-sided counterparts (i.e., negative values reflect right-dominant symptoms, positive values indicate left-dominant ones). Moreover, we observed the well-hypothesized relationship between symptom laterality on traditional clinical evaluations of motor impairment, which suggests that individuals with right-lateralized symptoms had more severe general, item-specific and bradykinesia-related impairments than individuals with left-lateralized symptoms. Nevertheless, future studies interrogating larger samples and hand kinematics from both body halves will be beneficial to confirm the impact of symptom laterality in PwP. Finally, it is important to note that while the current study included a cohort of PwP currently undergoing pharmacological *and* surgical treatment (i.e., levodopa medication, STN-DBS) for bradykinesia to disentangle diverse symptom- (i.e., movement-type) *and* disease-related (i.e., medication or stimulation status) alterations in motor impairment, future work in earlier stages of the disease (e.g., prior to DBS implantation) and also including longitudinal follow ups will undoubtably improve the generalizability of our findings to the greater population of PwP as a whole. Nevertheless, our data suggest that quantitative characterization of motor symptoms from wearable sensor recordings, as assessed in the current study, may serve as effective targets for identifying and effectively distinguishing clinically-relevant fluctuations in motor impairment, such as varying therapeutic interventions. Moreover, the development of time-resolved (i.e., single-trial), quantitative behavioral markers of clinical outcomes may substantially improve upon current, albeit subjective and less-reliable, clinical evaluations of motor impairment^8,9^, and further, may translate well to future investigations whereby trial-to-trial variation in motoric fluctuations is required to more precisely optimize therapeutic outcomes as they are administered in real-time (e.g., closed-loop, adaptive DBS applications)^27,51–53^.

## Methods

### Patient demographics

Twenty PwP (M_age_ = 62.6 years old, 43–80 years old, 3 females) implanted with subthalamic deep brain stimulation (STN-DBS: i.e., Abbott Infinity DBS System, lead model: 6170, Abbott, Plano, Texas, USA) were recruited for this study from the Center for Movement Disorders and Neuromodulation at the University Hospital Düsseldorf. Exclusionary criteria included any medical illness affecting CNS function, any neurological or psychiatric disorder (except PD), severe depression (Beck Depression Inventory > 30), or cognitive impairment (Mini-Mental State Examination < 26). Participants were tested in the clinically-defined medication ON and OFF states. Of note, the medication OFF state required withdrawal of dopaminergic medication 12 h prior to study completion. In addition, PwP were also tested in the clinically-defined stimulation ON and OFF state (for DBS settings, see Table S1). For a comprehensive description of PD-relevant clinical information, see Table 1. The local ethics committee at Heinrich Heine University Düsseldorf approved at the study (No. 2019-626_2). All patients provided written informed consent. All methods were performed in accordance with the Declaration of Helsinki.

**Table 1:** **Patient demographics and clinical metrics.**

Demographics (Mean ± SD)	
N	20
Age (yrs)	62.8 $\pm$ 8.3
Sex (% males)	85.7
Time since diagnosis (yrs)	12.9 $\pm$ 6.1
Time since DBS implantation (yrs)	3.1 $\pm$ 1.4
Symptom subtype (% akinetic-rigid dominant)	90.4
Symptom laterality index	− 0.47 $\pm$ 3.0
UPDRS Med OFF, Stim OFF	41.6 $\pm$ 11.7
UPDRS Med ON, Stim OFF	30.3 $\pm$ 12.8
UPDRS Med OFF, Stim ON	24.9 $\pm$ 10.0
UPDRS Med ON, Stim ON	14.4 $\pm$ 7.9

Symptom subtype was calculated using a ratio score of mean tremor item-specific sub-scores (i.e., Items 3.15–3.18) to mean bradykinesia item-specific sub-scores (i.e., Items 3.4–3.8, Item 3.14) from the MDS-UPDRS Part-III Motor Examination. If zero was in the numerator, PwP would be classified as akinetic-rigid dominant subtypes. If zero was in the denominator, PwP would be classified as tremor dominant symptom subtypes. Ratios greater than 1.5 indicate tremor dominant subtypes. Symptom laterality indices were calculated for each medication and stimulation status by subtracting the sum score of right-sided bradykinesia items (i.e., Items 3.4–3.8) from the left-sided ones per med/stim condition, with negative values reflecting right-dominance of symptom profiles and positive values reflecting left-dominance.

### Time-resolved analysis of behavior

Participants completed two standardized movement protocols which included a finger-tapping paradigm (i.e., Item 3.4 of UPDRS Part-III Examination) and pronation-supination rotational movement paradigm (i.e., Item 3.6 of UPDRS Part-III Examination) in accordance with the MDS-UPDRS recommendations^7^. Specifically, participants were instructed to complete ~ 10 s of consecutive movements as largely, quickly and precisely as possible, following initial instruction from the experienced MDS-UPDRS rater (RKS, BHB). PwP completed finger-tapping and rotational movement protocols with their hand raised in the air and a triaxial accelerometer (ADXL335 iMEMS Accelerometer, Analog Devices Inc., Norwood, MA, USA) attached to the right index finger^54^. Of note, as this data was collected as part of a larger DFG-funded project (TRR295—424778381) which utilized left-lateralized STN-DBS protocols during behavioral (i.e., accelerometer-based finger tapping and pronation-supination movements) and neurophysiological (i.e., magnetoencephalography) recordings to reduce the large ferromagnetic artifacts induced by DBS cables and generators implanted on the right body half, behavioral data was only recorded from the right hand. Simultaneously, we applied monopolar DBS of the left STN at therapeutically beneficial settings (i.e., 130 Hz, 60 µs pulse width, current therapeutic contact height, ≥ clinically-effective stimulation amplitude; Table S1), albeit participants also completed each movement protocol when STN-DBS was turned OFF. Together, each participant completed the aforementioned behavioral testing in the following medication and stimulation states: (1) medication OFF, stimulation OFF, (2) medication ON, stimulation OFF, (3) medication OFF, stimulation ON, (4) medication ON, stimulation ON—yielding a total of 80 observations in the dataset. Finally, all participants underwent full UPDRS Part-III testing in each clinically-defined medication/stimulation state.

In order to quantify finger-tapping and rotational movement metrics on the single-trial level, we developed a novel event detection algorithm using custom-written scripts in MATLAB (Version 2021a)^25^. Finger-tapping and rotational movement blocks were epoched (i.e., ~ 10 s of consecutive movements) and pre-processed. The acceleration signal was visually inspected for artifacts and filtered using a third order high-pass Butterworth filter (1 Hz cut-off frequency). Next, probable movement events (i.e., taps or rotations) were detected at the single-trial level using a two-stage approach. First, probable movement events were identified using a fixed-threshold algorithm based on the magnitude and jerk (i.e., rate of change of acceleration) percentile thresholds of the accelerometer vector (i.e., 90 and 95th percentiles for finger-tapping, respectively and 80 and 85th percentiles for rotational movements, respectively). The resulting time windows of probable finger-taps or pronation-supination rotations (i.e., time of movement onset to offset) were further confirmed using the *findpeaks* function in MATLAB (i.e., minimum peak prominence ≥ 2.5 SD above the accelerometer vector magnitude; minimum peak distance ≥ 100 ms), supplemented with visual inspection. The resulting confirmed finger-tapping and rotational movement events (i.e., single-trial movement onset to offset time windows) were then used to quantify single-trial and grand-averaged behavioral metrics pertinent to MDS-UPDRS rating recommendations including normalized general acceleration magnitude in m/s^2^ (normed to movement duration; i.e., movement onset to offset in ms), inter-movement interval or movement frequency (i.e., peak to peak distance in ms), movement smoothness (i.e., acceleration jerk in m/s^3^), and the coefficient of variation of each variable, reflecting the consistency in each metric across the finger-tapping and rotational movement block (for exemplary accelerometer traces during hand movements, see Fig. 1). Finally, improvements or decrements in acceleration magnitude (in m/s^2^) and movement pacing (i.e., inter-movement interval in ms) were quantified using the standardized regression coefficients (ß; i.e., slope) derived from linear mixed effects models (LMEs) of each kinematic feature as a function of trial number (fixed effect continuous variable), with subject included as a random effect. Of note, negative regression coefficients for acceleration magnitude (in m/s^2^) over time are reflective of general decrements in movement across time elapsed on the task, while positive regression coefficients for movement frequency (in ms) denote decrements in inter-movement intervals (i.e., movement pacing) as time elapsed on the task.

### Statistical analysis

First, in order to evaluate the influence of relevant clinical statuses (i.e., medication and stimulation status) on time-resolved features of fine hand movements, the aforementioned single-trial behavioral metrics were subjected to LMEs of medication and stimulation status (fixed effect factor with 4 levels), with subject and trial number included as a nested random effect. All LME analyses were conducted using the *lme4* package in R (Version 4.0.3). Importantly, all LME post-hoc analyses were corrected for multiple comparisons using Tukey’s multiple comparison test from the *emmeans* package in R.

Next, we aimed to evaluate the predictive capacity of time-resolved, quantitative behavioral outcomes on standard clinical evaluations of motor impairment (i.e., total and task-specific UPDRS outcomes). To index relevant clinical features of finger-tapping and rotational movement profiles in the current sample, we conducted exploratory factor analyses (EFA) to define a single component of movement using a compilation of accelerometer metrics exhibiting significant alterations as a function of clinical status (i.e., general acceleration magnitude, movement frequency, movement smoothness, coefficient of variation of each metric, slope of acceleration magnitude over time, slope of movement frequency over time) for finger-tapping and rotational movement data, separately. Essentially, we began with a set list of eight measures and progressively removed individual variables based on poor loadings (λ < 0.70), and overall model fit. Criteria for good model fit included a non-statistically significant chi square, a root mean squared error approximation (RMSEA) < 0.06, a comparative fit index (CFI) > 0.95, and a standardized root mean squared residual (SRMR) < 0.08 based on standards in the literature^55^. The best fitting model was used to define a latent variable for which a movement profile score (i.e., finger-tapping and rotational movement profile score, separately) was extracted per participant. Modeling and component extraction was completed using *lavaan* and *principal* functions in R, respectively. As such, movement profile scores were subsequently extracted per patient and entered as a fixed effect (continuous variable), with subject as a random effect in our LME of standard UPDRS outcomes (i.e., total UPDRS Part-III score, Item 3.4 sub-score, Item 3.6 sub-score) as a function of movement profile scores. Importantly, observations with UPDRS-rated sub-scores of 4 (N = 1) were omitted from the reported analyses, as this rating traditionally denotes the patient’s inability to complete the motor task ^7^, albeit our results held regardless of this exclusion. Of note, lower finger-tapping and rotational movement profile scores as presented herein are reflective of smoother, more controlled fine hand movements based on the directionality observed during the application of clinically-effective medication and stimulation therapies (i.e., greater acceleration magnitude, increased movement frequency, greater movement smoothness, lower movement metric variability, positive slopes for acceleration magnitude over time, negative slopes for movement frequency over time; see Figures S1, S2). Furthermore, as prior work suggests that symptom laterality (i.e., left or right-dominant symptom profiles) is related to the severity of disease progression, as well as overall prognoses of PD^32–34^, we included a continuous symptom laterality index^56^ as a control variable in our LMEs of finger tapping and rotational movement profiles predicting total, item-specific and bradykinesia-specific sum scores. Essentially, in order to identify left- or right-dominance of symptoms, scores from each item that contained a right and left-sided component related to bradykinesia symptoms (i.e., Item 3.4 finger tapping, Item 3.5 hand movements, Item 3.6 pronation supination movements, Item 3.7 toe tapping, Item 3.8 leg agility) were extracted for each individual. Symptom asymmetry was calculated using each participant’s motor sub-scores by subtracting the total symptom score from the right side from the total symptom sub-score from the left side (i.e., left-sided bradykinesia sum score—right-sided bradykinesia sum score). Negative values of symptom asymmetry indicate right-dominant symptom profiles, while positive values indicate left-lateralized symptoms.

Finally, as the presence of bradykinesia is pertinent for probable PD diagnoses and this predominant symptom profile was prevalent in > 90% of our patient cohort, we aimed to evaluate the differential predictive capacity of diverse hand movement profiles (i.e., finger-tapping movement profile scores, rotational movement profile scores, and their interaction) on bradykinesia-specific symptoms controlling for symptom laterality using LMEs in R. Specifically, a right-sided bradykinesia UPDRS score (comprising the sum of Item 3.4, 3.5, 3.6, 3.7, 3.8 sub-scores) was used as a dependent variable in our analysis.

### Supplementary Information

Supplementary Information.

## Data Availability

The anonymized data from this study will be made available to investigators upon reasonable request to the corresponding authors.

## References

[1] DeMaagd G, Philip A (2015). Parkinson’s disease and its management. P T.

[2] Greenland JC, Williams-Gray CH, Barker RA (2019). The clinical heterogeneity of Parkinson’s disease and its therapeutic implications. Eur. J. Neurosci..

[3] Hindle JV (2010). Ageing, neurodegeneration and Parkinson’s disease. Age Ageing.

[4] Valcour V (2008). Aging exacerbates extrapyramidal motor signs in the era of highly active antiretroviral therapy. J. NeuroVirol..

[5] Robinson-Papp J (2020). Characteristics of motor dysfunction in longstanding human immunodeficiency virus. Clin. Infect. Dis..

[6] Huebl J (2015). Bradykinesia induced by frequency-specific pallidal stimulation in patients with cervical and segmental dystonia. Parkinsonism Relat. Disord..

[7] Goetz CG (2008). Movement disorder society-sponsored revision of the unified Parkinson’s disease rating scale (MDS-UPDRS): Scale presentation and clinimetric testing results. Mov. Disord..

[8] Fereshtehnejad S-M, Postuma RB (2017). Subtypes of Parkinson’s disease: What do they tell us about disease progression?. Curr. Neurol. Neurosci. Rep..

[9] Nutt JG (2016). Motor subtype in Parkinson’s disease: Different disorders or different stages of disease?. Mov. Disord..

[10] Teshuva I (2019). Using wearables to assess bradykinesia and rigidity in patients with Parkinson’s disease: A focused, narrative review of the literature. J. Neural Transm..

[11] Stamatakis J (2013). Finger tapping clinimetric score prediction in Parkinson’s disease using low-cost accelerometers. Comput. Intell. Neurosci..

[12] Bobić V (2019). An expert system for quantification of bradykinesia based on wearable inertial sensors. Sensors.

[13] di Biase L (2018). Quantitative analysis of bradykinesia and rigidity in Parkinson’s disease. Front. Neurol..

[14] Delrobaei M, Tran S, Gilmore G, McIsaac K, Jog M (2016). Characterization of multi-joint upper limb movements in a single task to assess bradykinesia. J. Neurol. Sci..

[15] Lin, Z., Dai, H., Null, X., Yongsheng, null, Xia, X., null & Horng, S.-J., null. Quantification assessment of bradykinesia in Parkinson’s disease based on a wearable device. *Annu Int Conf IEEE Eng Med Biol Soc* **2017**, 803–806 (2017). 10.1109/EMBC.2017.8036946 29059994

[16] Summa S (2017). Assessing bradykinesia in Parkinson’s disease using gyroscope signals. IEEE Int. Conf. Rehabil. Robot.

[17] Lee CY (2016). A validation study of a smartphone-based finger tapping application for quantitative assessment of bradykinesia in Parkinson’s disease. PLoS One.

[18] Rabelo AG (2017). Objective assessment of bradykinesia estimated from the wrist extension in older adults and patients with Parkinson’s disease. Ann. Biomed. Eng..

[19] Eskofier BM (2016). Recent machine learning advancements in sensor-based mobility analysis: Deep learning for Parkinson’s disease assessment. Annu. Int. Conf. IEEE Eng. Med. Biol. Soc..

[20] Dai H, Lin H, Lueth TC (2015). Quantitative assessment of parkinsonian bradykinesia based on an inertial measurement unit. Biomed. Eng. Online.

[21] Kim J-W (2011). Quantification of bradykinesia during clinical finger taps using a gyrosensor in patients with Parkinson’s disease. Med. Biol. Eng. Comput..

[22] Keijsers NLW, Horstink MWIM, Gielen SCAM (2006). Ambulatory motor assessment in Parkinson’s disease. Mov. Disord..

[23] Griffiths RI (2012). Automated assessment of bradykinesia and dyskinesia in Parkinson’s disease. J. Parkinsons Dis..

[24] Rodríguez-Molinero A (2017). Analysis of correlation between an accelerometer-based algorithm for detecting Parkinsonian gait and UPDRS subscales. Front. Neurol..

[25] Spooner RK, Bahners BH, Schnitzler A, Florin E (2023). DBS-evoked cortical responses index optimal contact orientations and motor outcomes in Parkinson’s disease. npj Parkinsons Dis..

[26] Kühn AA (2008). High-frequency stimulation of the subthalamic nucleus suppresses oscillatory β activity in patients with Parkinson’s disease in parallel with improvement in motor performance. J. Neurosci..

[27] Hirschmann J, Schoffelen JM, Schnitzler A, van Gerven MAJ (2017). Parkinsonian rest tremor can be detected accurately based on neuronal oscillations recorded from the subthalamic nucleus. Clin. Neurophysiol..

[28] Timmermann L (2003). The cerebral oscillatory network of parkinsonian resting tremor. Brain.

[29] Jankovic J (1990). Variable expression of Parkinson’s disease: A base-line analysis of the DATATOP cohort. Parkinson Study Group Neurol..

[30] Jankovic J (2008). Parkinson’s disease: Clinical features and diagnosis. J. Neurol. Neurosurg. Psychiatry.

[31] Williams-Gray CH (2013). The CamPaIGN study of Parkinson’s disease: 10-year outlook in an incident population-based cohort. J. Neurol. Neurosurg. Psychiatry.

[32] Baumann CR, Held U, Valko PO, Wienecke M, Waldvogel D (2014). Body side and predominant motor features at the onset of Parkinson’s disease are linked to motor and nonmotor progression. Mov. Disord,.

[33] Frazzitta G (2015). Differences in muscle strength in parkinsonian patients affected on the right and left side. PLoS One.

[34] Munhoz RP (2013). Long-duration Parkinson’s disease: Role of lateralization of motor features. Parkinsonism Relat. Disord,.

[35] Sano Y (2016). Quantifying Parkinson’s disease finger-tapping severity by extracting and synthesizing finger motion properties. Med. Biol. Eng. Comput..

[36] Djurić-Jovičić M (2016). Finger tapping analysis in patients with Parkinson’s disease and atypical Parkinsonism. J. Clin. Neurosci..

[37] Habets J (2023). A first methodological development and validation of ReTap: An open-source UPDRS finger tapping assessment tool based on accelerometer-data. Sensors.

[38] Heinrichs-Graham E (2014). Neuromagnetic evidence of abnormal movement-related beta desynchronization in Parkinson’s disease. Cereb. Cortex.

[39] Brown P (2003). Oscillatory nature of human basal ganglia activity: Relationship to the pathophysiology of Parkinson’s disease. Mov. Disord..

[40] Cassidy M (2002). Movement-related changes in synchronization in the human basal ganglia. Brain.

[41] Hirschmann J (2011). Distinct oscillatory STN-cortical loops revealed by simultaneous MEG and local field potential recordings in patients with Parkinson’s disease. NeuroImage.

[42] Spooner RK, Wilson TW (2022). Cortical theta-gamma coupling governs the adaptive control of motor commands. Brain Commun..

[43] Bahners BH, Spooner RK, Hartmann CJ, Schnitzler A, Florin E (2023). Subthalamic stimulation evoked cortical responses relate to motor performance in Parkinson’s disease. Brain Stimul..

[44] Lofredi R (2023). Pallidal beta activity is linked to stimulation-induced slowness in dystonia. Mov. Disord..

[45] Stegemöller EL (2015). Repetitive finger movement performance differs among Parkinson’s disease, progressive supranuclear palsy, and spinocerebellar ataxia. J. Clin. Mov. Disord..

[46] Spooner RK (2023). Mitochondrial redox environments predict sensorimotor brain-behavior dynamics in adults with HIV. Brain Behav. Immun..

[47] Krismer F (2022). The unified multiple system atrophy rating scale: Status, critique, and recommendations. Mov. Disord..

[48] Teixeira AL, Maia DP, Cardoso F (2005). UFMG Sydenham’s chorea rating scale (USCRS): Reliability and consistency. Mov. Disord..

[49] Piot I (2020). The progressive supranuclear palsy clinical deficits scale. Mov. Disord..

[50] Aggarwal A, Aggarwal N, Nagral A, Jankharia G, Bhatt M (2009). A novel Global assessment scale for Wilson’s disease (GAS for WD). Mov. Disord..

[51] Beudel M, Brown P (2016). Adaptive deep brain stimulation in Parkinson’s disease. Parkinsonism Relat. Disord..

[52] Chen R (2022). Clinical neurophysiology of Parkinson’s disease and parkinsonism. Clin. Neurophysiol. Pract..

[53] Neumann W-J (2019). Toward electrophysiology-based intelligent adaptive deep brain stimulation for movement disorders. Neurotherapeutics.

[54] Piitulainen H, Bourguignon M, De Tiège X, Hari R, Jousmäki V (2013). Coherence between magnetoencephalography and hand-action-related acceleration, force, pressure, and electromyogram. Neuroimage.

[55] Hu L, Bentler P (1999). Cutoff criteria for fit indexes in covariance structure analysis: Conventional criteria versus new alternatives. Struct. Equ. Model. Multidiscip. J..

[56] Heinrichs-Graham E, Santamaria PM, Gendelman HE, Wilson TW (2017). The cortical signature of symptom laterality in Parkinson’s disease. Neuroimage Clin..

