# Supplementary material for: Time-resolved quantification of fine hand movements as a proxy for evaluating bradykinesia-induced motor dysfunction
Source: Sci Rep. 2024 Mar 4;14:5340. doi: 10.1038/s41598-024-55862-4 (PMC10912452; doi:10.1038/s41598-024-55862-4)
Supplement: Supplementary file 1 — Supplementary Information. [file 41598_2024_55862_MOESM1_ESM.docx]

**Supplementary Materials**

**Table S1. DBS Parameter Settings for Accelerometer Recordings**

| **subID** | **Contact** | **Frequency (Hz)** | **Pulse Width (μs)** | **Amplitude (mA)** |
| --- | --- | --- | --- | --- |
| sub-004 | C | 130 | 60 | 1 |
| sub-006 | A | 130 | 60 | 3 |
| sub-007 | A | 130 | 60 | 2 |
| sub-008 | OMNI | 130 | 60 | 1 |
| sub-009 | OMNI | 130 | 60 | 3 |
| sub-010 | C | 130 | 60 | 2 |
| sub-011 | B | 130 | 60 | 2 |
| sub-013 | A | 130 | 60 | 2 |
| sub-014 | B | 130 | 60 | 2 |
| sub-015 | C | 130 | 60 | 3 |
| sub-016 | B | 130 | 60 | 2 |
| sub-017 | OMNI | 130 | 60 | 3 |
| sub-019 | OMNI | 130 | 60 | 3 |
| sub-020 | A | 130 | 60 | 3 |
| sub-021 | C | 130 | 60 | 2 |
| sub-022 | OMNI | 130 | 60 | 4 |
| sub-023 | OMNI | 130 | 60 | 2 |
| sub-024 | OMNI | 130 | 60 | 4 |
| sub-025 | OMNI | 130 | 60 | 4 |
| sub-026 | OMNI | 130 | 60 | 4 |

*Note.* Left STN DBS settings for monopolar review sessions of right-handed finger movements.

**Table S2. Model results of clinical status on single-trial finger-tapping acceleration metrics.**

| ***Acceleration Magnitude*** | | | | | |
| --- | --- | --- | --- | --- | --- |
| **Effect** | **F** | **p** | **Effect Size (d)** | **Lower CI** | **Upper CI** |
| Clinical Status | 31.37 | **0.001*** | 0.32 | 0.21 | 0.44 |
| ***Post Hoc Testing*** | | | | | |
| **Main Effect of Clinical Status** | **t** | **p** | **Effect Size (d)** | **Lower CI** | **Upper CI** |
| 1 vs. 2 | -1.73 | 0.307 | -0.06 | -0.12 | 0.01 |
| 1 vs. 3 | -1.97 | 0.199 | -0.06 | -0.13 | 0.00 |
| 1 vs. 4 | -8.98 | **0.001*** | -0.29 | -0.36 | -0.23 |
| 2 vs. 3 | 0.06 | 0.999 | 0.00 | -0.06 | 0.07 |
| 2 vs. 4 | -6.32 | **0.001*** | -0.20 | -0.27 | -0.14 |
| 3 vs. 4 | -7.40 | **0.001*** | -0.24 | -0.30 | -0.18 |
| ***Estimated Marginal Means (EMM)*** | | | | | |
| **Experimental Session** | | **EMM (m/s^2^)** | **SE** | **Lower CI** | **Upper CI** |
| 1: Med OFF, Stim OFF | | 7.83 | 0.74 | 6.28 | 9.37 |
| 2: Med ON, Stim OFF | | 8.15 | 0.75 | 6.60 | 9.70 |
| 3: Med OFF, Stim ON | | 8.14 | 0.74 | 6.60 | 9.68 |
| 4: Med ON, Stim ON | | 9.32 | 0.74 | 7.78 | 10.86 |
| ***Coefficient of Variation in Acceleration Magnitude*** | | | | | |
| **Effect** | **F** | **p** | **Effect Size (d)** | **Lower CI** | **Upper CI** |
| Clinical Status | 65.74 | **0.001*** | 0.46 | 0.34 | 0.57 |
| ***Post Hoc Testing*** | | | | | |
| **Main Effect of Clinical Status** | **t** | **p** | **Effect Size (d)** | **Lower CI** | **Upper CI** |
| 1 vs. 2 | 3.40 | **0.004** | 0.11 | 0.05 | 0.17 |
| 1 vs. 3 | 9.56 | **0.001*** | 0.30 | 0.24 | 0.37 |
| 1 vs. 4 | 13.08 | **0.001*** | 0.42 | 0.35 | 0.48 |
| 2 vs. 3 | 4.77 | **0.001*** | 0.15 | 0.09 | 0.21 |
| 2 vs. 4 | 8.32 | **0.001*** | 0.26 | 0.20 | 0.33 |
| 3 vs. 4 | 4.15 | **0.001*** | 0.13 | 0.07 | 0.19 |
| ***Estimated Marginal Means (EMM)*** | | | | | |
| **Experimental Session** | | **EMM (%)** | **SE** | **Lower CI** | **Upper CI** |
| 1: Med OFF, Stim OFF | | 20.70 | 1.46 | 17.66 | 23.70 |
| 2: Med ON, Stim OFF | | 18.40 | 1.50 | 15.29 | 21.40 |
| 3: Med OFF, Stim ON | | 15.20 | 1.45 | 12.18 | 18.20 |
| 4: Med ON, Stim ON | | 12.80 | 1.46 | 9.77 | 15.80 |

*Note*. Cohen’s d effect sizes and associated 95% confidence intervals (CI) were calculated based on appropriate test statistics using the *effectsize* package in R. Bolded values indicate statistical significance and trending effects based on *p_corrected_* < .05 and .10, respectively following Tukey’s correction for multiple comparisons and non-overlapping confidence intervals of effect sizes. Clinical status designations: (1) stim OFF/med OFF, (2) stim OFF/med ON, (3) stim ON/med OFF, (4) stim ON/med ON. **p_corrected_* < .001.

**Table S3. Model results of clinical status on single-trial finger-tapping pacing metrics.**

| ***Inter-tap Interval (i.e., tapping pace)*** | | | | | |
| --- | --- | --- | --- | --- | --- |
| **Effect** | **F** | **p** | **Effect Size (d)** | **Lower CI** | **Upper CI** |
| Clinical Status | 25.96 | **0.001*** | 0.29 | 0.18 | 0.40 |
| ***Post Hoc Testing*** | | | | | |
| **Main Effect of Clinical Status** | **t** | **p** | **Effect Size (d)** | **Lower CI** | **Upper CI** |
| 1 vs. 2 | 2.74 | **0.032** | 0.09 | 0.03 | 0.15 |
| 1 vs. 3 | 7.24 | **0.001*** | 0.23 | 0.17 | 0.30 |
| 1 vs. 4 | 7.71 | **0.001*** | 0.25 | 0.18 | 0.31 |
| 2 vs. 3 | 3.43 | **0.004** | 0.11 | 0.05 | 0.17 |
| 2 vs. 4 | 4.17 | **0.001*** | 0.13 | 0.07 | 0.20 |
| 3 vs. 4 | 0.89 | 0.811 | 0.03 | -0.03 | 0.09 |
| ***Estimated Marginal Means (EMM)*** | | | | | |
| **Experimental Session** | | **EMM (ms)** | **SE** | **Lower CI** | **Upper CI** |
| 1: Med OFF, Stim OFF | | 564.00 | 40.70 | 480.00 | 648.00 |
| 2: Med ON, Stim OFF | | 504.00 | 42.20 | 418.00 | 590.00 |
| 3: Med OFF, Stim ON | | 430.00 | 40.40 | 347.00 | 513.00 |
| 4: Med ON, Stim ON | | 413.00 | 40.70 | 330.00 | 497.00 |
| ***Coefficient of Variation in Inter-tap Interval*** | | | | | |
| **Effect** | **F** | **p** | **Effect Size (d)** | **Lower CI** | **Upper Ci** |
| Clinical Status | 5.78 | **0.001*** | 0.14 | 0.03 | 0.25 |
| ***Post Hoc Testing*** | | | | | |
| **Main Effect of Clinical Status** | **t** | **p** | **Effect Size (d)** | **Lower CI** | **Upper Ci** |
| 1 vs. 2 | 1.13 | 0.671 | 0.04 | -0.03 | 0.10 |
| 1 vs. 3 | 3.63 | **0.002** | 0.12 | 0.05 | 0.18 |
| 1 vs. 4 | 3.36 | **0.005** | 0.11 | 0.05 | 0.17 |
| 2 vs. 3 | 1.96 | 0.203 | 0.06 | 0.00 | 0.13 |
| 2 vs. 4 | 1.87 | 0.241 | 0.06 | 0.00 | 0.12 |
| 3 vs. 4 | -0.09 | 0.999 | 0.00 | -0.07 | 0.06 |
| ***Estimated Marginal Means (EMM)*** | | | | | |
| **Experimental Session** | | **EMM (%)** | **SE** | **Lower CI** | **Upper CI** |
| 1: Med OFF, Stim OFF | | 42.40 | 5.24 | 31.60 | 53.10 |
| 2: Med ON, Stim OFF | | 39.00 | 5.44 | 27.90 | 50.10 |
| 3: Med OFF, Stim ON | | 33.20 | 5.18 | 22.60 | 43.90 |
| 4: Med ON, Stim ON | | 33.50 | 5.22 | 22.70 | 44.20 |

*Note*. Cohen’s d effect sizes and associated 95% confidence intervals (CI) were calculated based on appropriate test statistics using the *effectsize* package in R. Bolded values indicate statistical significance and trending effects based on *p_corrected_* < .05 and .10, respectively following Tukey’s correction for multiple comparisons and non-overlapping confidence intervals of effect sizes. Clinical status designations: (1) stim OFF/med OFF, (2) stim OFF/med ON, (3) stim ON/med OFF, (4) stim ON/med ON. **p_corrected_* < .001.

**Table S4. Model results of clinical status on single-trial finger-tapping movement smoothness metrics.**

| ***Acceleration Jerk (i.e., movement execution smoothness)*** | | | | | |
| --- | --- | --- | --- | --- | --- |
| **Effect** | **F** | **p** | **Effect Size (d)** | **Lower CI** | **Upper CI** |
| Clinical Status | 6.59 | **0.001*** | 0.15 | 0.03 | 0.26 |
| ***Post Hoc Testing*** | | | | | |
| **Main Effect of Clinical Status** | **t** | **p** | **Effect Size (d)** | **Lower CI** | **Upper CI** |
| 1 vs. 2 | -1.25 | 0.599 | -0.04 | -0.10 | 0.02 |
| 1 vs. 3 | -1.25 | 0.593 | -0.04 | -0.10 | 0.02 |
| 1 vs. 4 | -4.27 | **0.001*** | -0.14 | -0.20 | -0.08 |
| 2 vs. 3 | 0.18 | 0.998 | 0.00 | -0.06 | 0.07 |
| 2 vs. 4 | -2.65 | **0.041** | -0.09 | -0.15 | -0.02 |
| 3 vs. 4 | -3.25 | **0.007** | -0.11 | -0.17 | -0.04 |
| ***Estimated Marginal Means (EMM)*** | | | | | |
| **Experimental Session** | | **EMM (m/s^2^)** | **SE** | **Lower CI** | **Upper CI** |
| 1: Med OFF, Stim OFF | | 0.0445 | 0.00458 | 0.0350 | 0.0539 |
| 2: Med ON, Stim OFF | | 0.0474 | 0.00472 | 0.0377 | 0.0570 |
| 3: Med OFF, Stim ON | | 0.0469 | 0.00454 | 0.0376 | 0.0563 |
| 4: Med ON, Stim ON | | 0.0536 | 0.00458 | 0.0441 | 0.0630 |
| ***Coefficient of Variation in Acceleration Jerk*** | | | | | |
| **Effect** | **F** | **p** | **Effect Size (d)** | **Lower CI** | **Upper Ci** |
| Clinical Status | 17.48 | **0.001*** | 0.23 | 0.12 | 0.34 |
| ***Post Hoc Testing*** | | | | | |
| **Main Effect of Clinical Status** | **t** | **p** | **Effect Size (d)** | **Lower CI** | **Upper Ci** |
| 1 vs. 2 | -2.92 | **0.019** | -0.09 | -0.15 | -0.03 |
| 1 vs. 3 | 2.79 | **0.027** | 0.09 | 0.03 | 0.15 |
| 1 vs. 4 | 4.24 | **0.001*** | 0.13 | 0.07 | 0.20 |
| 2 vs. 3 | 5.35 | **0.001*** | 0.17 | 0.11 | 0.23 |
| 2 vs. 4 | 6.72 | **0.001*** | 0.21 | 0.15 | 0.28 |
| 3 vs. 4 | 1.66 | 0.346 | 0.05 | -0.01 | 0.11 |
| ***Estimated Marginal Means (EMM)*** | | | | | |
| **Experimental Session** | | **EMM (%)** | **SE** | **Lower CI** | **Upper CI** |
| 1: Med OFF, Stim OFF | | 81.70 | 9.61 | 62.30 | 101.20 |
| 2: Med ON, Stim OFF | | 108.50 | 10.63 | 87.20 | 129.80 |
| 3: Med OFF, Stim ON | | 60.20 | 9.34 | 41.20 | 79.10 |
| 4: Med ON, Stim ON | | 47.20 | 9.61 | 27.70 | 66.60 |

*Note*. Cohen’s d effect sizes and associated 95% confidence intervals (CI) were calculated based on appropriate test statistics using the *effectsize* package in R. Bolded values indicate statistical significance and trending effects based on *p_corrected_* < .05 and .10, respectively following Tukey’s correction for multiple comparisons and non-overlapping confidence intervals of effect sizes. Clinical status designations: (1) stim OFF/med OFF, (2) stim OFF/med ON, (3) stim ON/med OFF, (4) stim ON/med ON. **p_corrected_* < .001.

**Table S5. Model results of clinical status on finger tapping movement acceleration magnitude over time (i.e., slope of acceleration improvements/decrements).**

| ***Slope of Acceleration Magnitude*** | | | | | |
| --- | --- | --- | --- | --- | --- |
| **Effect** | **F** | **p** | **Effect Size (d)** | **Lower CI** | **Upper CI** |
| Clinical Status | 2.68 | **0.057** | 0.46 | -0.10 | 1.01 |
| ***Post Hoc Testing*** | | | | | |
| **Main Effect of Clinical Status** | **t** | **p** | **Effect Size (d)** | **Lower CI** | **Upper CI** |
| 1 vs. 2 | -0.19 | 0.998 | -0.03 | -0.29 | 0.24 |
| 1 vs. 3 | 1.93 | 0.227 | 0.27 | 0.00 | 0.54 |
| 1 vs. 4 | 1.93 | 0.227 | 0.27 | 0.00 | 0.54 |
| 2 vs. 3 | 1.94 | 0.224 | 0.28 | 0.00 | 0.55 |
| 2 vs. 4 | 1.94 | 0.224 | 0.28 | 0.00 | 0.55 |
| 3 vs. 4 | 0.00 | 1.000 | 0.00 | -0.27 | 0.27 |
| ***Estimated Marginal Means (EMM)*** | | | | | |
| **Experimental Session** | | **EMM (ß)** | **SE** | **Lower CI** | **Upper CI** |
| 1: Med OFF, Stim OFF | | 0.11 | 0.09 | -0.07 | 0.29 |
| 2: Med ON, Stim OFF | | 0.14 | 0.11 | -0.07 | 0.35 |
| 3: Med OFF, Stim ON | | -0.12 | 0.09 | -0.30 | 0.06 |
| 4: Med ON, Stim ON | | -0.12 | 0.09 | -0.30 | 0.05 |

*Note*. Cohen’s d effect sizes and associated 95% confidence intervals (CI) were calculated based on appropriate test statistics using the *effectsize* package in R. Bolded values indicate statistical significance and trending effects based on *p_corrected_* < .05 and .10, respectively following Tukey’s correction for multiple comparisons and non-overlapping confidence intervals of effect sizes. Clinical status designations: (1) stim OFF/med OFF, (2) stim OFF/med ON, (3) stim ON/med OFF, (4) stim ON/med ON.

**Table S6. Model results of clinical status on finger tapping movement pacing over time (i.e., slope of inter-tap interval improvements/decrements).**

| ***Slope of Inter-tap Interval (i.e., Tapping Pace)*** | | | | | |
| --- | --- | --- | --- | --- | --- |
| **Effect** | **F** | **p** | **Effect Size (d)** | **Lower CI** | **Upper CI** |
| Clinical Status | 1.28 | 0.292 | 0.32 | -0.24 | 0.87 |
| ***Post Hoc Testing*** | | | | | |
| **Main Effect of Clinical Status** | **t** | **p** | **Effect Size (d)** | **Lower CI** | **Upper CI** |
| 1 vs. 2 | -0.96 | 0.772 | -0.14 | -0.41 | 0.14 |
| 1 vs. 3 | -1.79 | 0.292 | -0.26 | -0.53 | 0.02 |
| 1 vs. 4 | -0.41 | 0.977 | -0.06 | -0.33 | 0.21 |
| 2 vs. 3 | -0.65 | 0.914 | -0.09 | -0.37 | 0.18 |
| 2 vs. 4 | 0.58 | 0.937 | 0.08 | -0.19 | 0.36 |
| 3 vs. 4 | 1.34 | 0.540 | 0.19 | -0.08 | 0.47 |
| ***Estimated Marginal Means (EMM)*** | | | | | |
| **Experimental Session** | | **EMM (ß)** | **SE** | **Lower CI** | **Upper CI** |
| 1: Med OFF, Stim OFF | | -0.102 | 0.07 | -0.24 | 0.04 |
| 2: Med ON, Stim OFF | | 0.005 | 0.08 | -0.17 | 0.16 |
| 3: Med OFF, Stim ON | | 0.062 | 0.07 | -0.08 | 0.20 |
| 4: Med ON, Stim ON | | -0.064 | 0.07 | -0.21 | 0.08 |

*Note*. Cohen’s d effect sizes and associated 95% confidence intervals (CI) were calculated based on appropriate test statistics using the *effectsize* package in R. Bolded values indicate statistical significance and trending effects based on *p_corrected_* < .05 and .10, respectively following Tukey’s correction for multiple comparisons and non-overlapping confidence intervals of effect sizes. Clinical status designations: (1) stim OFF/med OFF, (2) stim OFF/med ON, (3) stim ON/med OFF, (4) stim ON/med ON.

**Table S7. Model results of clinical status on single-trial rotational movement acceleration metrics.**

| ***Acceleration Magnitude*** | | | | | |
| --- | --- | --- | --- | --- | --- |
| **Effect** | **F** | **p** | **Effect Size (d)** | **Lower CI** | **Upper CI** |
| Clinical Status | 94.77 | **0.001*** | 0.63 | 0.50 | 0.76 |
| ***Post Hoc Testing*** | | | | | |
| **Main Effect of Clinical Status** | **t** | **p** | **Effect Size (d)** | **Lower CI** | **Upper CI** |
| 1 vs. 2 | -10.20 | **0.001*** | -0.37 | -0.44 | -0.30 |
| 1 vs. 3 | -12.55 | **0.001*** | -0.46 | -0.53 | -0.38 |
| 1 vs. 4 | -15.15 | **0.001*** | -0.55 | -0.63 | -0.47 |
| 2 vs. 3 | -0.68 | 0.906 | -0.02 | -0.10 | 0.05 |
| 2 vs. 4 | -3.89 | **0.001*** | -0.14 | -0.21 | -0.07 |
| 3 vs. 4 | -3.33 | **0.005** | -0.12 | -0.19 | -0.05 |
| ***Estimated Marginal Means (EMM)*** | | | | | |
| **Experimental Session** | | **EMM (m/s^2^)** | **SE** | **Lower CI** | **Upper CI** |
| 1: Med OFF, Stim OFF | | 4.67 | 0.63 | 0.37 | 5.96 |
| 2: Med ON, Stim OFF | | 6.42 | 0.63 | 5.11 | 7.72 |
| 3: Med OFF, Stim ON | | 6.54 | 0.63 | 5.24 | 7.84 |
| 4: Med ON, Stim ON | | 7.10 | 0.63 | 5.80 | 8.40 |
| ***Coefficient of Variation in Acceleration Magnitude*** | | | | | |
| **Effect** | **F** | **p** | **Effect Size (d)** | **Lower CI** | **Upper CI** |
| Clinical Status | 14.13 | **0.001*** | 0.24 | 0.11 | 0.36 |
| ***Post Hoc Testing*** | | | | | |
| **Main Effect of Clinical Status** | **t** | **p** | **Effect Size (d)** | **Lower CI** | **Upper CI** |
| 1 vs. 2 | 4.61 | **0.001*** | 0.16 | 0.09 | 0.23 |
| 1 vs. 3 | 5.39 | **0.001*** | 0.19 | 0.12 | 0.26 |
| 1 vs. 4 | 5.02 | **0.001*** | 0.18 | 0.11 | 0.25 |
| 2 vs. 3 | 0.12 | 0.999 | 0.00 | -0.07 | 0.07 |
| 2 vs. 4 | 0.04 | 1.000 | 0.00 | -0.07 | 0.07 |
| 3 vs. 4 | -0.09 | 0.999 | 0.00 | -0.07 | 0.07 |
| ***Estimated Marginal Means (EMM)*** | | | | | |
| **Experimental Session** | | **EMM (%)** | **SE** | **Lower CI** | **Upper CI** |
| 1: Med OFF, Stim OFF | | 17.30 | 1.11 | 15.00 | 19.60 |
| 2: Med ON, Stim OFF | | 14.20 | 1.16 | 11.80 | 16.60 |
| 3: Med OFF, Stim ON | | 14.10 | 1.13 | 11.80 | 16.40 |
| 4: Med ON, Stim ON | | 14.20 | 1.12 | 11.90 | 16.50 |

*Note*. Cohen’s d effect sizes and associated 95% confidence intervals (CI) were calculated based on appropriate test statistics using the *effectsize* package in R. Bolded values indicate statistical significance and trending effects based on *p_corrected_* < .05 and .10, respectively following Tukey’s correction for multiple comparisons and non-overlapping confidence intervals of effect sizes. Clinical status designations: (1) stim OFF/med OFF, (2) stim OFF/med ON, (3) stim ON/med OFF, (4) stim ON/med ON. **p_corrected_* < .001.

**Table S8. Model results of clinical status on single-trial rotational movement pacing metrics.**

| ***Inter-rotation Interval (i.e., rotation pace)*** | | | | | |
| --- | --- | --- | --- | --- | --- |
| **Effect** | **F** | **p** | **Effect Size (d)** | **Lower CI** | **Upper CI** |
| Clinical Status | 2.54 | **0.056** | 0.10 | -0.02 | 0.23 |
| ***Post Hoc Testing*** | | | | | |
| **Main Effect of Clinical Status** | **t** | **p** | **Effect Size (d)** | **Lower CI** | **Upper CI** |
| 1 vs. 2 | -1.23 | 0.611 | -0.04 | -0.12 | 0.03 |
| 1 vs. 3 | 1.13 | 0.670 | 0.04 | -0.03 | 0.11 |
| 1 vs. 4 | -1.62 | 0.366 | -0.06 | -0.13 | 0.01 |
| 2 vs. 3 | 2.08 | 0.161 | 0.08 | 0.00 | 0.15 |
| 2 vs. 4 | -0.27 | 0.993 | 0.00 | -0.08 | 0.06 |
| 3 vs. 4 | -2.53 | **0.056** | -0.09 | -0.16 | -0.02 |
| ***Estimated Marginal Means (EMM)*** | | | | | |
| **Experimental Session** | | **EMM (ms)** | **SE** | **Lower CI** | **Upper CI** |
| 1: Med OFF, Stim OFF | | 431.00 | 32.10 | 366.00 | 496.00 |
| 2: Med ON, Stim OFF | | 465.00 | 35.40 | 394.00 | 536.00 |
| 3: Med OFF, Stim ON | | 403.00 | 33.20 | 336.00 | 471.00 |
| 4: Med ON, Stim ON | | 473.00 | 33.10 | 406.00 | 540.00 |
| ***Coefficient of Variation in Inter-rotation Interval*** | | | | | |
| **Effect** | **F** | **p** | **Effect Size (d)** | **Lower CI** | **Upper Ci** |
| Clinical Status | 2.23 | **0.084** | 0.1 | -0.03 | 0.22 |
| ***Post Hoc Testing*** | | | | | |
| **Main Effect of Clinical Status** | **t** | **p** | **Effect Size (d)** | **Lower CI** | **Upper Ci** |
| 1 vs. 2 | -1.96 | 0.204 | -0.07 | -0.14 | 0.00 |
| 1 vs. 3 | 0.7 | 0.899 | 0.03 | -0.05 | 0.10 |
| 1 vs. 4 | 0.16 | 0.999 | 0 | -0.07 | 0.08 |
| 2 vs. 3 | 2.41 | **0.076** | 0.09 | 0.02 | 0.16 |
| 2 vs. 4 | 2.07 | 0.165 | 0.08 | 0.00 | 0.15 |
| 3 vs. 4 | -0.47 | 0.965 | -0.02 | -0.09 | 0.05 |
| ***Estimated Marginal Means (EMM)*** | | | | | |
| **Experimental Session** | | **EMM (%)** | **SE** | **Lower CI** | **Upper CI** |
| 1: Med OFF, Stim OFF | | 71.00 | 7.33 | 56.00 | 85.90 |
| 2: Med ON, Stim OFF | | 82.30 | 7.96 | 66.30 | 98.40 |
| 3: Med OFF, Stim ON | | 67.40 | 7.52 | 52.10 | 82.70 |
| 4: Med ON, Stim ON | | 70.10 | 7.50 | 54.90 | 85.40 |

*Note*. Cohen’s d effect sizes and associated 95% confidence intervals (CI) were calculated based on appropriate test statistics using the *effectsize* package in R. Bolded values indicate statistical significance and trending effects based on *p_corrected_* < .05 and .10, respectively following Tukey’s correction for multiple comparisons and non-overlapping confidence intervals of effect sizes. Clinical status designations: (1) stim OFF/med OFF, (2) stim OFF/med ON, (3) stim ON/med OFF, (4) stim ON/med ON. **p_corrected_* < .001.

**Table S9. Model results of clinical status on single-trial rotational movement smoothness metrics.**

| ***Acceleration Jerk (i.e., movement execution smoothness)*** | | | | | |
| --- | --- | --- | --- | --- | --- |
| **Effect** | **F** | **p** | **Effect Size (d)** | **Lower CI** | **Upper CI** |
| Clinical Status | 2.72 | **0.043** | 0.11 | -0.02 | 0.23 |
| ***Post Hoc Testing*** | | | | | |
| **Main Effect of Clinical Status** | **t** | **p** | **Effect Size (d)** | **Lower CI** | **Upper CI** |
| 1 vs. 2 | -1.54 | 0.412 | -0.06 | -0.13 | 0.02 |
| 1 vs. 3 | -1.98 | 0.195 | -0.07 | -0.14 | 0.00 |
| 1 vs. 4 | -2.67 | **0.039** | -0.10 | -0.17 | -0.03 |
| 2 vs. 3 | -0.04 | 1.000 | 0.00 | -0.07 | 0.07 |
| 2 vs. 4 | -0.71 | 0.894 | -0.03 | -0.10 | 0.04 |
| 3 vs. 4 | -0.74 | 0.880 | -0.03 | -0.10 | 0.04 |
| ***Estimated Marginal Means (EMM)*** | | | | | |
| **Experimental Session** | | **EMM (m/s^2^)** | **SE** | **Lower CI** | **Upper CI** |
| 1: Med OFF, Stim OFF | | 0.00595 | 0.00089 | 0.00412 | 0.00778 |
| 2: Med ON, Stim OFF | | 0.00674 | 0.00094 | 0.00482 | 0.00866 |
| 3: Med OFF, Stim ON | | 0.00677 | 0.00090 | 0.00492 | 0.00861 |
| 4: Med ON, Stim ON | | 0.00711 | 0.00090 | 0.00526 | 0.00895 |
| ***Coefficient of Variation in Acceleration Jerk*** | | | | | |
| **Effect** | **F** | **p** | **Effect Size (d)** | **Lower CI** | **Upper Ci** |
| Clinical Status | 18.08 | **0.001*** | 0.27 | 0.15 | 0.4 |
| ***Post Hoc Testing*** | | | | | |
| **Main Effect of Clinical Status** | **t** | **p** | **Effect Size (d)** | **Lower CI** | **Upper Ci** |
| 1 vs. 2 | -7.17 | **0.001*** | -0.26 | -0.33 | -0.18 |
| 1 vs. 3 | -3.84 | **0.001*** | -0.14 | -0.21 | -0.07 |
| 1 vs. 4 | -4.24 | **0.001*** | -0.15 | -0.22 | -0.08 |
| 2 vs. 3 | 3.59 | **0.002** | 0.13 | 0.06 | 0.20 |
| 2 vs. 4 | 3.20 | **0.008** | 0.11 | 0.04 | 0.18 |
| 3 vs. 4 | -0.55 | 0.945 | -0.02 | -0.09 | 0.05 |
| ***Estimated Marginal Means (EMM)*** | | | | | |
| **Experimental Session** | | **EMM (%)** | **SE** | **Lower CI** | **Upper CI** |
| 1: Med OFF, Stim OFF | | 32.60 | 4.61 | 23.10 | 42.00 |
| 2: Med ON, Stim OFF | | 56.10 | 4.93 | 46.10 | 66.10 |
| 3: Med OFF, Stim ON | | 43.60 | 4.71 | 34.00 | 53.20 |
| 4: Med ON, Stim ON | | 45.40 | 4.69 | 35.80 | 55.00 |

*Note*. Cohen’s d effect sizes and associated 95% confidence intervals (CI) were calculated based on appropriate test statistics using the *effectsize* package in R. Bolded values indicate statistical significance and trending effects based on *p_corrected_* < .05 and .10, respectively following Tukey’s correction for multiple comparisons and non-overlapping confidence intervals of effect sizes. Clinical status designations: (1) stim OFF/med OFF, (2) stim OFF/med ON, (3) stim ON/med OFF, (4) stim ON/med ON. **p_corrected_* < .001.

**Table S10. Model results of clinical status on rotational movement acceleration magnitude over time (i.e., slope of acceleration improvements/decrements).**

| ***Slope of Acceleration Magnitude*** | | | | | |
| --- | --- | --- | --- | --- | --- |
| **Effect** | **F** | **p** | **Effect Size (d)** | **Lower CI** | **Upper CI** |
| Clinical Status | 0.25 | 0.863 | 0.14 | -0.42 | 0.70 |
| ***Post Hoc Testing*** | | | | | |
| **Main Effect of Clinical Status** | **t** | **p** | **Effect Size (d)** | **Lower CI** | **Upper CI** |
| 1 vs. 2 | -0.61 | 0.928 | -0.08 | -0.35 | 0.18 |
| 1 vs. 3 | -0.06 | 0.999 | 0.00 | -0.27 | 0.26 |
| 1 vs. 4 | 0.21 | 0.997 | 0.03 | -0.24 | 0.29 |
| 2 vs. 3 | 0.56 | 0.943 | 0.08 | -0.19 | 0.34 |
| 2 vs. 4 | 0.81 | 0.851 | 0.11 | -0.16 | 0.38 |
| 3 vs. 4 | 0.26 | 0.994 | 0.04 | -0.23 | 0.30 |
| ***Estimated Marginal Means (EMM)*** | | | | | |
| **Experimental Session** | | **EMM (ß)** | **SE** | **Lower CI** | **Upper CI** |
| 1: Med OFF, Stim OFF | | -0.14 | 0.08 | -0.31 | 0.03 |
| 2: Med ON, Stim OFF | | -0.07 | 0.10 | -0.26 | 0.13 |
| 3: Med OFF, Stim ON | | -0.14 | 0.08 | -0.31 | 0.03 |
| 4: Med ON, Stim ON | | -0.17 | 0.09 | -0.34 | 0.00 |

*Note*. Cohen’s d effect sizes and associated 95% confidence intervals (CI) were calculated based on appropriate test statistics using the *effectsize* package in R. Bolded values indicate statistical significance and trending effects based on *p_corrected_* < .05 and .10, respectively following Tukey’s correction for multiple comparisons and non-overlapping confidence intervals of effect sizes. Clinical status designations: (1) stim OFF/med OFF, (2) stim OFF/med ON, (3) stim ON/med OFF, (4) stim ON/med ON.

**Table S11. Model results of clinical status on rotational movement pacing over time (i.e., slope of inter-tap interval improvements/decrements).**

| ***Slope of Inter-rotation Interval (i.e., Rotation Pace)*** | | | | | |
| --- | --- | --- | --- | --- | --- |
| **Effect** | **F** | **p** | **Effect Size (d)** | **Lower CI** | **Upper CI** |
| Clinical Status | 0.24 | 0.867 | 0.12 | -0.37 | 0.61 |
| ***Post Hoc Testing*** | | | | | |
| **Main Effect of Clinical Status** | **t** | **p** | **Effect Size (d)** | **Lower CI** | **Upper CI** |
| 1 vs. 2 | 0.51 | 0.956 | 0.07 | -0.20 | 0.33 |
| 1 vs. 3 | 0.36 | 0.984 | 0.05 | -0.22 | 0.31 |
| 1 vs. 4 | 0.80 | 0.854 | 0.11 | -0.16 | 0.37 |
| 2 vs. 3 | -0.17 | 0.998 | -0.02 | -0.29 | 0.24 |
| 2 vs. 4 | 0.23 | 0.995 | 0.03 | -0.23 | 0.30 |
| 3 vs. 4 | 0.44 | 0.971 | 0.06 | -0.21 | 0.32 |
| ***Estimated Marginal Means (EMM)*** | | | | | |
| **Experimental Session** | | **EMM (ß)** | **SE** | **Lower CI** | **Upper CI** |
| 1: Med OFF, Stim OFF | | -0.04 | 0.14 | -0.33 | 0.24 |
| 2: Med ON, Stim OFF | | -0.15 | 0.17 | -0.48 | 0.18 |
| 3: Med OFF, Stim ON | | -0.12 | 0.14 | -0.40 | 0.17 |
| 4: Med ON, Stim ON | | -0.20 | 0.14 | -0.49 | 0.08 |

*Note*. Cohen’s d effect sizes and associated 95% confidence intervals (CI) were calculated based on appropriate test statistics using the *effectsize* package in R. Bolded values indicate statistical significance and trending effects based on *p_corrected_* < .05 and .10, respectively following Tukey’s correction for multiple comparisons and non-overlapping confidence intervals of effect sizes. Clinical status designations: (1) stim OFF/med OFF, (2) stim OFF/med ON, (3) stim ON/med OFF, (4) stim ON/med ON.

**Table S12.** **Achieved loadings derived from principal components analyses of finger-tapping dynamics.**

| ***Finger-Tapping Movement Profiles*** | | | |
| --- | --- | --- | --- |
| **Metric** | **Achieved**  **Loadings** | **Eigenvalue** | **Variance**  **Accounted For** |
| Acceleration Magnitude | 0.91 | 2.75 | 68.8% |
| Acceleration Variability | 0.85 |  |  |
| Acceleration Jerk | 0.82 |  |  |
| Jerk Variability | 0.73 |  |  |

*Note*. Behavioral metrics exhibiting significant alterations as a function of medication and stimulation status were subjected to principal components analyses to derive a single component of finger-tapping movement profiles. Reverse coded acceleration magnitude, reverse coded acceleration jerk (i.e., movement smoothness) and the coefficient of variation in each metric accounted for 68.8% of the variance in finger-tapping movement profiles.

**Table S13.** **Achieved loadings derived from principal components analyses of rotational movement dynamics.**

| ***Rotational Movement Profiles*** | | | |
| --- | --- | --- | --- |
| **Metric** | **Achieved**  **Loadings** | **Eigenvalue** | **Variance**  **Accounted For** |
| Acceleration Magnitude | 0.87 | 1.90 | 63.3% |
| Acceleration Jerk | 0.89 |  |  |
| Inter-rotation Interval | 0.59 |  |  |

*Note*. Behavioral metrics exhibiting significant alterations as a function of medication and stimulation status were subjected to principal components analyses to derive a single component of rotational movement profiles. Reverse coded acceleration magnitude, reverse coded acceleration jerk (i.e., movement smoothness) and inter-rotation interval (i.e., rotation pacing) accounted for 63.3% of the variance in rotational movement profiles.

**Table S14. Model results of clinical status on finger tapping movement profile scores.**

| ***Finger Tapping Movement Profile Score*** | | | | | |
| --- | --- | --- | --- | --- | --- |
| **Effect** | **F** | **p** | **Effect Size (d)** | **Lower CI** | **Upper CI** |
| Clinical Status | 3.49 | **0.022** | 0.52 | -0.04 | 1.08 |
| ***Post Hoc Testing*** | | | | | |
| **Main Effect of Clinical Status** | **t** | **p** | **Effect Size (d)** | **Lower CI** | **Upper CI** |
| 1 vs. 2 | -0.03 | 1.000 | 0.00 | -0.27 | 0.26 |
| 1 vs. 3 | 1.43 | 0.490 | 0.19 | -0.08 | 0.46 |
| 1 vs. 4 | 2.73 | **0.041** | 0.37 | 0.09 | 0.64 |
| 2 vs. 3 | 1.32 | 0.558 | 0.18 | -0.09 | 0.45 |
| 2 vs. 4 | 2.55 | **0.064** | 0.35 | 0.07 | 0.62 |
| 3 vs. 4 | 1.35 | 0.535 | 0.18 | -0.09 | 0.45 |
| ***Estimated Marginal Means (EMM)*** | | | | | |
| **Experimental Session** | | **EMM (ß)** | **SE** | **Lower CI** | **Upper CI** |
| 1: Med OFF, Stim OFF | | 0.03 | 0.04 | -0.05 | 0.11 |
| 2: Med ON, Stim OFF | | 0.03 | 0.04 | -0.05 | 0.12 |
| 3: Med OFF, Stim ON | | -0.02 | 0.04 | -0.10 | 0.06 |
| 4: Med ON, Stim ON | | -0.07 | 0.04 | -0.14 | 0.01 |

*Note*. Cohen’s d effect sizes and associated 95% confidence intervals (CI) were calculated based on appropriate test statistics using the *effectsize* package in R. Bolded values indicate statistical significance and trending effects based on *p_corrected_* < .05 and .10, respectively following Tukey’s correction for multiple comparisons and non-overlapping confidence intervals of effect sizes. Clinical status designations: (1) stim OFF/med OFF, (2) stim OFF/med ON, (3) stim ON/med OFF, (4) stim ON/med ON.

**Table S15. Model results of clinical status on rotational movement tapping movement profile scores.**

| ***Rotational Movement Profile Score*** | | | | | |
| --- | --- | --- | --- | --- | --- |
| **Effect** | **F** | **p** | **Effect Size (d)** | **Lower CI** | **Upper CI** |
| Clinical Status | 1.61 | 0.200 | 0.36 | -0.20 | 0.92 |
| ***Post Hoc Testing*** | | | | | |
| **Main Effect of Clinical Status** | **t** | **p** | **Effect Size (d)** | **Lower CI** | **Upper CI** |
| 1 vs. 2 | 1.22 | 0.619 | 0.17 | -0.10 | 0.44 |
| 1 vs. 3 | 1.25 | 0.601 | 0.17 | -0.10 | 0.44 |
| 1 vs. 4 | 2.10 | 0.166 | 0.29 | 0.01 | 0.56 |
| 2 vs. 3 | -0.09 | 0.999 | -0.01 | -0.28 | 0.26 |
| 2 vs. 4 | 0.74 | 0.879 | 0.10 | -0.17 | 0.37 |
| 3 vs. 4 | 0.89 | 0.812 | 0.12 | -0.15 | 0.39 |
| ***Estimated Marginal Means (EMM)*** | | | | | |
| **Experimental Session** | | **EMM (ß)** | **SE** | **Lower CI** | **Upper CI** |
| 1: Med OFF, Stim OFF | | 0.08 | 0.08 | -0.09 | 0.24 |
| 2: Med ON, Stim OFF | | -0.03 | 0.09 | -0.20 | 0.15 |
| 3: Med OFF, Stim ON | | -0.02 | 0.08 | -0.18 | 0.14 |
| 4: Med ON, Stim ON | | -0.09 | 0.08 | -0.25 | 0.07 |

*Note*. Cohen’s d effect sizes and associated 95% confidence intervals (CI) were calculated based on appropriate test statistics using the *effectsize* package in R. Bolded values indicate statistical significance and trending effects based on *p_corrected_* < .05 and .10, respectively following Tukey’s correction for multiple comparisons and non-overlapping confidence intervals of effect sizes. Clinical status designations: (1) stim OFF/med OFF, (2) stim OFF/med ON, (3) stim ON/med OFF, (4) stim ON/med ON.


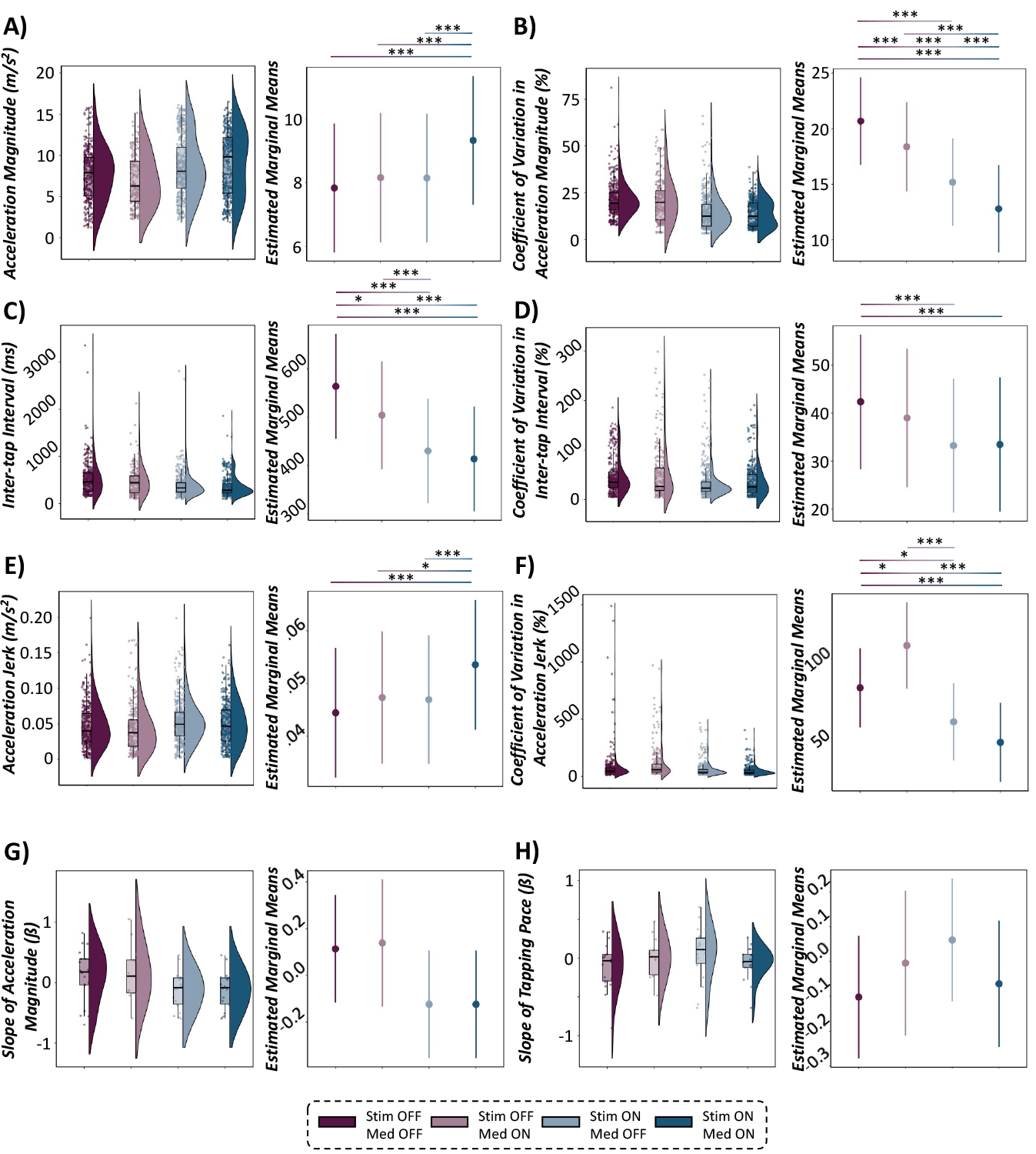


**Figure S1. Quantitative Finger-Tapping Metrics Index Medication and DBS Stimulation Status in Parkinson’s Disease.** (Left panels A-H): Quantitative metrics of finger-tapping identified using fixed threshold algorithm (i.e., percentile thresholds of root mean square, jerk) of triaxial accelerometer data on the right index finger during standard UPDRS tapping protocols. Raincloud plots include a combined box plot (box edges: first 25th percentile quartile to third 75th percentile quartile; center line: median; data minima/maxima: whisker length), histogram distribution and individual scatter points of each accelerometer metric. (Right panels A-H): Estimated marginal means and 95% confidence intervals of each kinematic feature from LMEs as a function of therapeutic status. Post hoc significance is fixed for left and right panels (A-H) and color gradients from light to dark and purple to blue denote significant post hoc effects across each medication and stimulation state. **p_corr_* < .05, ***p_corr_* < .01, ****p_corr_* < .005. For more details regarding post-hoc analyses, see Tables S2-6.


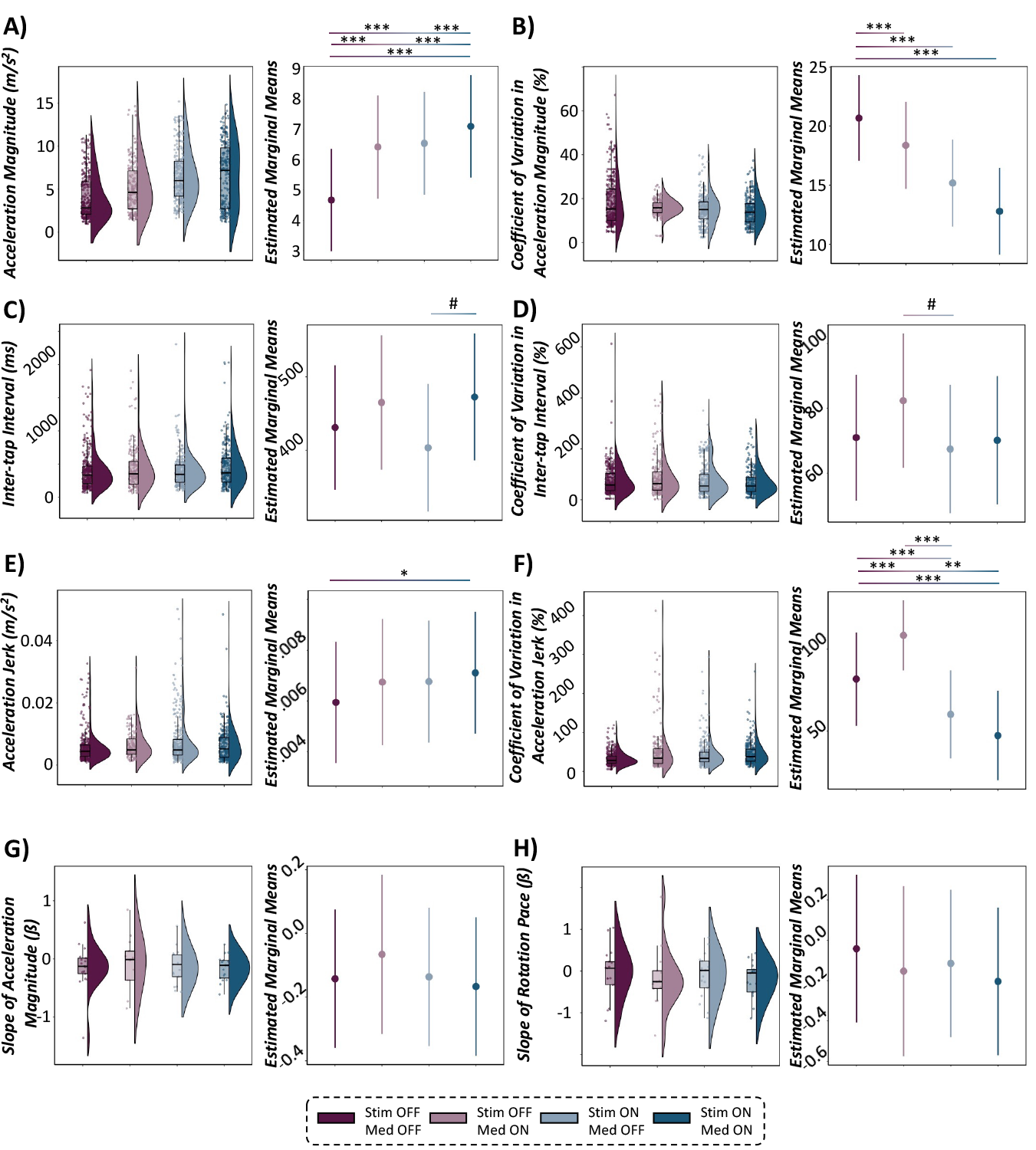


**Figure S2. Quantitative Rotational Movement Metrics Index Medication and DBS Stimulation Status in Parkinson’s Disease.** (Left panels A-H): Quantitative metrics of finger tapping identified using fixed threshold algorithm (i.e., percentile thresholds of root mean square, jerk) of triaxial accelerometer data on the right index finger during standard UPDRS tapping protocols. Raincloud plots include a combined box plot (box edges: first 25th percentile quartile to third 75th percentile quartile; center line: median; data minima/maxima: whisker length), histogram distribution and individual scatter points of each accelerometer metric. (Right panels A-H): Estimated marginal means and 95% confidence intervals of each kinematic feature from LMEs as a function of therapeutic status. Post hoc significance is fixed for left and right panels (A-H) and color gradients from light to dark and purple to blue denote significant post hoc effects across each medication and stimulation state. ^#^*p_corr_* < .10, **p_corr_* < .05, ***p_corr_* < .01, ****p_corr_* < .005. For more details regarding post-hoc analyses, see Tables S7-11.
